# Cell-to-Cell Interactions and Signals Involved in the Reconstitution of Peripheral CD8^+^ T_CM_ and T_EM_ Cell Pools

**DOI:** 10.1371/journal.pone.0017423

**Published:** 2011-03-14

**Authors:** Bruno Zaragoza, César Evaristo, Adrien Kissenpfennig, Valentina Libri, Bernard Malissen, Benedita Rocha, António A. Freitas, Afonso R. M. Almeida

**Affiliations:** 1 Lymphocyte Population Biology Unit, CNRS, URA 1961, Institut Pasteur, Paris, France; 2 INSERM, U591, Institut Federatif de Recherche 94, Université Paris-Descartes, Paris, France; 3 INSERM, U631, CNRS, UMR6102, Centre d'Immunologie de Marseille-Luminy, Université de la Méditerrannée, Marseille, France; 4 U. Signalisation des cytokines, CNRS, Institut Pasteur, Paris, France; University Paris Sud, France

## Abstract

We here describe novel aspects of CD8^+^ and CD4^+^ T cell subset interactions that may be clinically relevant and provide new tools for regulating the reconstitution of the peripheral CD8^+^ T cell pools in immune-deficient states. We show that the reconstitution capacity of transferred isolated naïve CD8^+^ T cells and their differentiation of effector functions is limited, but both dramatically increase upon the co-transfer of CD4^+^ T cells. This helper effect is complex and determined by multiple factors. It was directly correlated to the number of helper cells, required the continuous presence of the CD4^+^ T cells, dependent on host antigen-presenting cells (APCs) expressing CD40 and on the formation of CD4/CD8/APC cell clusters. By comparing the recovery of (CD44^+^CD62L^high^) T_CM_ and (CD44^+^CD62L^low^) T_EM_ CD8^+^ T cells, we found that the accumulation of T_CM_ and T_EM_ subsets is differentially regulated. T_CM_-cell accumulation depended mainly on type I interferons, interleukin (IL)-6, and IL-15, but was independent of CD4^+^ T-cell help. In contrast, T_EM_-cell expansion was mainly determined by CD4^+^ T-cell help and dependent on the expression of IL-2Rβ by CD8 cells, on IL-2 produced by CD4^+^ T-cells, on IL-15 and to a minor extent on IL-6.

## Introduction

Clinical peripheral T cell lymphopenia is common following infectious diseases, such as, HIV, or aggressive therapies for neoplasia and autoimmune diseases. The capacity to recover peripheral T cell numbers, which is a hallmark of T cell homeostasis, raises interesting possibilities for the rehabilitation of such immune-deficient states. Mature peripheral T cells, once transferred into a lymphopenic environment, expand considerably and can repopulate the peripheral T cell pool [1]. Such capacity for lymphopenia driven proliferation (LDP) has been shown to be dependent on both T cell receptor (TCR)-major histocompatibility complex (MHC) interactions [2], [3] and cytokines [4], [5], [6]. However, though LDP is often considered to be a homeostatic response, it may incapable of reconstituting the peripheral immune system [1], [7] as present in a normal individual. Different T cell clones show divergent proliferation capacities [8]: therefore only a limited fraction of the transferred cells expand [1] resulting in reducing T cell repertoires [9]. Moreover, restoration of the peripheral T cell pool modifies the functional ability of lymphocytes [7], [10], [11] and in some cases may cause self-aggressive pathologies [9], [12], [13]. These observations imply that a full recovery of immune competence is not necessarily achieved through the recovery of cell numbers: to maintain immune responsiveness, discrete lymphocytes subpopulations that confer different qualities to the immune system must also be maintained [14] including naïve CD4^+^ and CD8^+^ T cells, memory CD8^+^T_CM_ and CD8^+^T_EM_ subpopulations [15] CD4^+^ T_regs_, and TH17 CD4^+^ effector T cells [12], [16], [17], [18]. In addition, T cell homeostasis and immune responses are the result of a number of dynamic interactions between different T cell populations and the environment and amongst themselves [7], [19]. For example, CD4^+^ and CD8^+^ T cells are known to interact to generate CD8^+^ T cell memory during immune responses and to confer protective functions to CD8^+^ T cells during homeostatic proliferation [20]. The presence of CD4^+^ T cells greatly impacts the number and quality of CD8^+^ “memory” T cells generated during immune responses either directly through cell-contact dependent CD40-CD40L interactions [21] or indirectly through third party populations like dendritic cells (DCs) [22]. All these populations are expected to coexist in physiological settings: thus, it is important to establish how interactions occur between the co-expanding T cell populations and how they contribute to the restoration of the CD8^+^ T cell subpopulations following lymphopenia.

We investigated the cellular interactions that occur after adoptive transfer of isolated T cell populations into T cell deficient hosts.

## Methods

### Ethics Statement

Mice were cared for in accordance with Pasteur Institute guidelines in compliance with European animal welfare regulations, and all animal studies were approved by the Pasteur Institute Safety Committee in accordance with French and European guidelines and by the ethics Committee of Paris 1 (permits 2010-0002, 2010-0003 and 2010-0004).

### Mice

C57Bl/6.Ly5^b^ and C57Bl/6.Ly5^a^ mice were purchased from Charles Rivers (France). B6.129-*Lat* ^tm6Mal^ mice were derived by Dr. B. Malissen [23]. B6.CCR5^−/−^, B6.Rag2^−/−^IL-15^−/−^, B6.IL-2Rβ^−/−^ and B6.IFNAR^−/−^ were gifts from Drs. C. Combadière, J. Di Santo, C. Surh, and M. Albert and the B6.IL-15^−/−^ mice were from Taconic Europe (Denmark). All mice including B6.CD3ε^−/−^, B6.Rag2^−/−^, B6.CD40^−/−^, B6.Rag2^−/−^CD40^−/−^, B6.IL-2^−/−^, B6.CD3ε^−/−^IL-2^−/−^, B6.IL-6^−/−^ and B6.Rag2^−/−^IL-6^−/−^ were kept in our animal facilities.

### Cell transfer

Lymph node (LN) cells from donor mice were enriched for CD4^+^ or CD8^+^ T cells by Dynal MPC6 magnetic cell sorting (Dynal, Oslo) or auto MACS (Miltenyi-Biotec, Bergisch Gladbach, Germany). After selection >90% of the remaining population was CD4^+^ or CD8^+^. These cells labeled with combinations of anti-CD4 (L3T4/RM4-5), anti-CD45RB and anti-CD25 (7D4), or anti-CD8, anti-CD44 and anti-CD62L antibodies were sorted using a FacsAria (Becton Dickinson, San Jose, CA USA). The purity of the sorted CD44^+^CD62L^+^CD8^+^, CD44^+^CD62L^−^CD8^+^, CD44^−^CD62L^+^CD8^+^, CD45RB^high^CD25^−^CD4^+^ and CD45RB^low^CD25^+^CD4^+^ populations was >96%. To estimate cell division donor CD8^+^ T cells were labeled with carboxyfluorescein diacetate succinimidyl ester (CFSE) (Molecular Probes) as previously described [24]. Immune-deficient hosts were intravenously injected with purified LN CD4^+^ or CD8^+^ T cells alone or mixed and sacrificed at varying time intervals thereafter. Spleen and inguinal and mesenteric LN suspensions were prepared and the number and phenotype of the cells evaluated. Mice with different Ly5 allotypes allowed different donor cells to be discriminated. The total peripheral T cells represented the number of cells recovered in the host's spleen plus twice the number of the inguinal and mesenteric LN cells. To deplete Lat-DTR CD4^+^ T cells, host mice received five intraperitoneal injections of 1 µg of diphtheria toxin (DT) (List Biological Laboratories, Campbell, CA) on alternating days.

### Flow cytometry

The following monoclonal antibodies were used: anti-CD45.1, anti-CD45.2, anti-CD3ε (145-2C11), anti-CD4 (L3T4/RM4-5), anti-CD8, anti-CD11c, anti-CD25 (7D4), anti-CD44 (IM7), anti CD45RB (16A), anti-CD62L (MEL14), anti-CD69 (H1.2F3), anti-TCRβ (H57), anti-IFNγ and anti-TNFα (MP6-XT22), from Pharmingen (San Diego, CA, USA); anti-CD44 (IM781) and anti-CD62L (MEL14) from Caltag (San Francisco, CA, USA); and anti-CD25 from Southern Biotechnologies. CCR7 staining was performed using the ELC.Fc fusion protein. Four/six color staining used the appropriate combinations of FITC, PE, TRI-color, PerCP, PECy7, biotin, APC, AlexaFluor647 and APCCy7-coupled antibodies. Biotin-coupled antibodies were secondarily labeled with APC-, TRI-Color- (Caltag, San Francisco, CA, USA), PerCP- (Becton Dickinson, San Jose, CA, USA) or APCCy7-coupled (Pharmingen) streptavidin. Dead cells were excluded based on light-scattering. All data acquisition and analyses were performed with a FACSCanto (Becton Dickinson, San Jose, CA USA) interfaced with Macintosh CellQuest or FloJo software. To estimate cell division in vivo, mice received two daily intraperitoneal injections of 1 mg 5-ethynyl-2′-deoxyuridine (EdU) at 12-hour interval for three consecutive days. EdU incorporation was detected using a Click-iT EdU flow cytometry kit (Invitrogen). In vivo cell death was detected by staining with Annexin V (BD Biosciences).

### Single-cell multiple parametric quantitative RT-PCR

RT-PCR was performed as previously described [25]. To ensure that each well contained a T cell, CD3ε mRNA was amplified simultaneously with other genes. The mRNAs studied were TGFβ1 (*Tgfβ1*), TNF-α (*Tnf*), IFNγ (*Ifng*), Perforin (*Perf)*, Granzyme A (*Gzma)*, Granzyme B (*Gzmb)*, FasL (*Fasl)*, CCR7 (*Rccr7)*, IL-7R (*Il7r*), IL-10R (*Il10r*), IL-15R (*Il15r*), IL-21R (*Il21r*), IL-2 (*Il2*), IL-15 (*Il15*), IL-21 (*Il21*), TGFβRI (*Tgfbr1*), TGFβRII (*Tgfbr2*), TGFβRIII (*Tgfbr3*) and CD3ε. These single-cell studies revealed considerable cell-to-cell variation [25]. Results were expressed as positive (mRNA detected) or negative (mRNA absent).

### Statistical analysis

Sample means were compared using the unpaired Students' *t* test. In cases of considerably sample variances, Welsh's correction was used. For linear regression analysis, the Spearman correlation test was used. Sample means were considered significantly different at *P*<0.05.

## Results

### CD4^+^ T cells modify CD8^+^ T cell LDP

**Figure 1:**
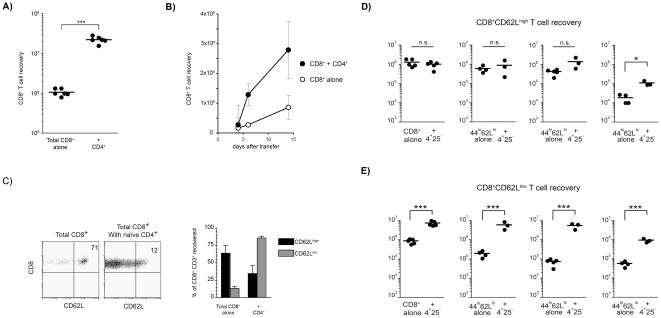
CD4^+^ T cells modify the LDP of CD8^+^ T cells. (**A**) 2×10^4^ CD8^+^ T cells were transferred alone or co-transferred with 2×10^4^ CD4^naive^ T cells into CD3ε^−/−^ lymphopenic mice. The data represent the absolute numbers of CD8^+^ T cells recovered 8 weeks after transfer in each group of mice with each dot representing recovery in an individual host and the bar indicating the mean values in each group. The presence of CD4^+^ T cells significantly increased CD8^+^ T cell recoveries (***p<0.001). Similar findings were obtained in 5–6 independent experiments. (**B**) The number of CD8^+^ T cells recovered at 4, 6 and 14 days after when transferred (alone, open dots) or co-transferred with CD4^naive^ T cells (filled dots) into CD3ε^−/−^ lymphopenic mice. Data represent mean±se. n = 3 mice. Similar results were obtained in a second independent experiment. (**C**) Dot plots showing the CD62L phenotype of CD8^+^ T cells recovered 10 weeks after the transfer of 2×10^4^ CD8^+^ T cells alone (left) or with 2×10^4^ CD4^naive^ T cells (right) into lymphopenic mice. Each dot plot shows a representative staining and values inside the dot plot are the % in the respective quadrant. The right hand graph shows the relative representation of the CD62L^high^ and CD62L^low^ cells (mean±se) among the CD8^+^ T cells recovered when transferred alone or in the presence of CD4^+^ T cells. Similar results were obtained in 5–6 independent experiments. (**D**) Absolute numbers of CD62L^high^CD8^+^ T_CM_ cells recovered 10 weeks after the injection of 2×10^4^ total CD8^+^ T cell from different subpopulations transferred alone or with 2×10^4^ CD4^+^ T cells into CD3ε^−/−^ hosts. (**E**) Absolute numbers of CD62L^low^CD8^+^ T_EM_ cells recovered 10 weeks after the injection of 2×10^4^ of different CD8^+^ T cell from different subpopulations transferred alone or with 2×10^4^ CD4^+^ T cells into CD3ε^−/−^ hosts. Statistically significant differences are shown (*p<0.05, **p<0.01, ***p<0.001). In all cases CD8^+^ T_EM_ cell numbers increased 10-fold or more in presence of CD4 help. Please note that recovery of CD62L^high^ cells after the transfer of CD62L^low^CD8^+^ cells in presence of CD4 help was increased, but this recovery was 10-fold lower (10^5^ cells) than the recovery of the CD8^+^ T_EM_ cells.

CD8^+^ T cells are capable of considerable expansion after transfer to T cell-deficient hosts: 4 to 8 weeks after the transfer of 2×10^4^ purified mature CD8^+^ T cells into CD3ε^−/−^ mice we recovered ∼10^6^ CD8^+^ T cells. However, upon the co-transfer of CD4^+^ T cells, the CD8^+^ T cells expanded to about 10 to 30-fold higher numbers (>10^7^) revealing that CD4^+^ T cells play a major role in promoting CD8^+^ T cell expansion (Figure 1A). This effect was observed early after transfer and persisted throughout peripheral reconstitution (Figure 1B). An evaluation of CD8^+^ T cell division showed that, 4 days after transfer, the presence of CD4^+^ T cells increased the fraction of CFSE-labeled CD8^+^ T cells with more than 5–6 divisions (Figure S1). By 2 or 4 weeks the CD8^+^ T cells co-transferred with CD4^+^ T cells contained a higher fraction of EdU^+^ cells (Figure S1) demonstrating that help improved cell division even at later time points. CD4^+^ T cell co-transfer also reduced the percentage of Annexin V^+^ CD8^+^ T cells (Figure S1). These results demonstrate that CD4^+^ T cells help in the peripheral expansion of CD8^+^ T cells by enhancing proliferation and reducing cell death.

Next we characterized the CD8^+^ T cell populations generated in the absence or presence of CD4-help. In the absence of CD4^+^ T cells, 75–80% of the recovered CD8^+^ T cells were CD62L^high^, and CD4-help lead to the accumulation of cells with a CD62L^low^CCR7^low^ phenotype (Figure 1C; Figure S1). To investigate whether this effect affected all CD8^+^ T cell subsets, we sorted naïve (CD44^lo^CD62L^high^), T_CM_ (CD44^high^CD62L^high^), and T_EM_ (CD44^high^CD62L^low^) CD8^+^ T cells from WT donors and transferred 2×10^4^ of each cell subset into different groups of CD3ε^−/−^ hosts, either alone or with identical numbers of CD4^+^ T cells. The co-transfer of CD4^+^ T cells resulted in a strong helper effect on all CD8^+^ T cell subtypes. Surprisingly, when we characterized the CD8^+^ T cells recovered in the hosts, we found that CD4^+^ T cell help did not significantly modify the recovery of CD8^+^CD62L^high^ T cells (Figure 1D), but had a major effect on the accumulation of cells with the CD62L^low^ T_EM_ phenotype (Figure 1E). It should be pointed out that after transfer of CD62L^low^CD8^+^ cells in the presence of CD4 cells the recovery of CD62L^high^ cells was increased, but this recovery was 10-fold lower (10^5^ cells) than the recovery of the CD8^+^ T_EM_ cells, which increased 10–30 times to over 10^6^ cells. Overall, these results demonstrated that CD4^+^ T cell help promotes the accumulation of CD8^+^CD62L^low^ T cells and suggest that during CD8^+^ T cell recovery the relative representation of the two cellular subsets may be modified according to the environmental conditions.

### CD4^+^ T cells promote accumulation of differentiated CD8^+^ T_EM_ cells

**Figure 2:**
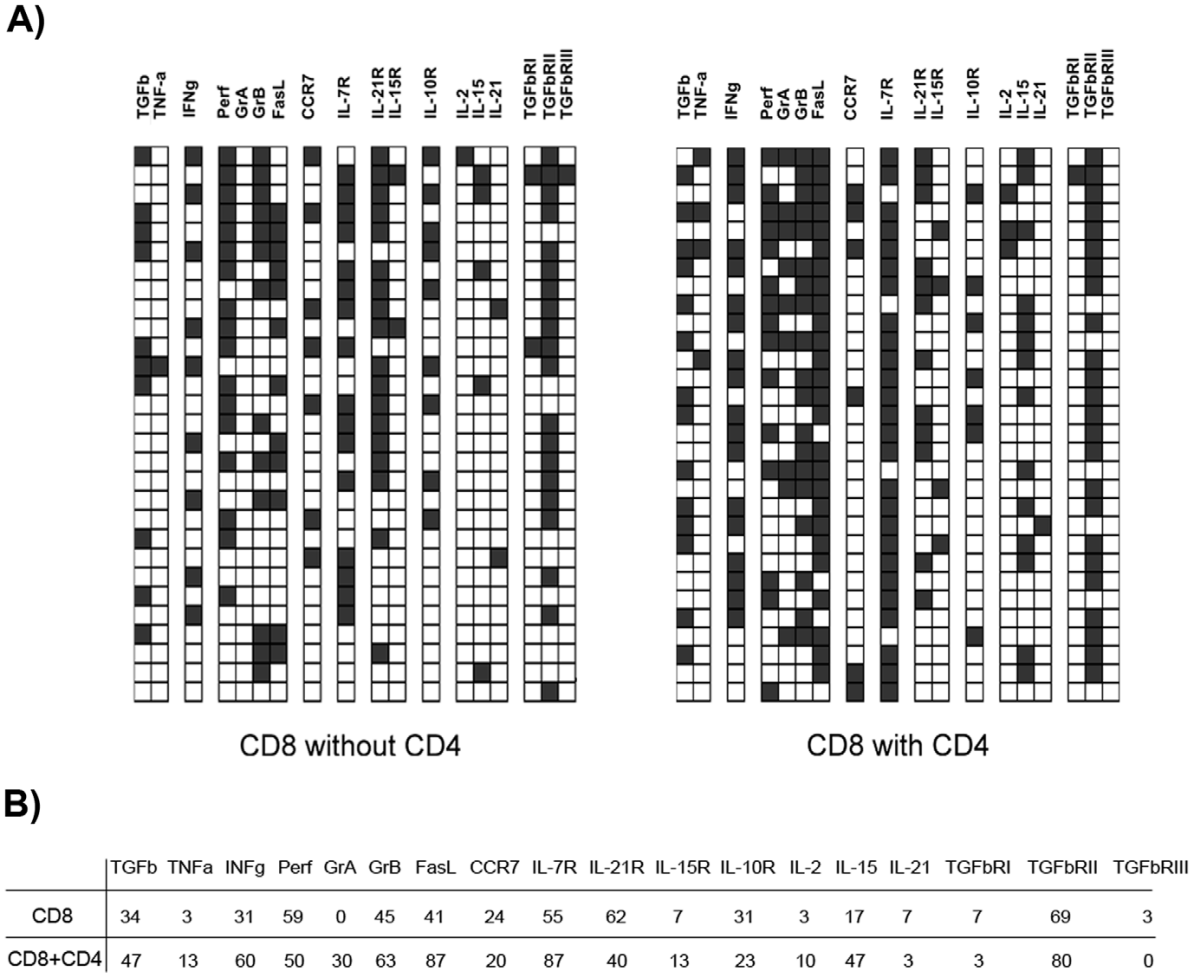
The help effect selectively expands fully differentiated T_EM_ CD8^+^ T cells. (**A**) 1 to 2×10^4^ CD8^+^ T cells were transferred alone or with 2×10^4^ CD4^+^ T cells into CD3ε^−/−^ hosts. Mice were sacrificed 8 weeks after the transfer and CD8^+^CD44^high^CD62L^low^ cells from the lymph node were single-cell sorted. Multiplex RT-PCR of 19 genes was performed on 32 single-cells for the two groups. For each individual cell, black squares indicate that mRNA gene expression was detected; white squares indicate that the mRNA for the corresponding gene was absent ( = 0) [28]. (**B**) The table shows the percent of cells positive for each gene for the two groups of cells detailed in (A). Cytotoxic cells require co-expression of both Perforin and Granzymes A and B [27]. These cells could only be found among “helped” CD8^+^ T cells. Please note that “helped” CD8^+^ T cells harbored a significant fraction of cells expressing *Il2* and *Il15* mRNAs, which are very rarely expressed during antigen-specific responses and the expression of which is absent in memory T cells [28]. “Helped” CD8^+^ T cell populations also showed a higher frequency of *Il7r*-expressing and a lower frequency of *Il21r* expressing cells.

We characterized the expanded CD8^+^ T cells by comparing the co-expression of multiple genes for CD8^+^ T cell function, in individual CD8^+^CD62L^low^ T cells recovered from mice co-injected or not injected with CD4^+^ T cells. We studied the gene expression profiles directly in single ex-vivo cells to prevent the bias introduced by in vitro re-stimulation [26]. The frequency of cells expressing IFNγ, granzyme A and B, and FasL effector molecules mRNAs was much higher among the “helped” CD8^+^CD62L^low^ T cells (Figure 2). Individual CD8^+^ T cells co-expressing perforin and both granzymes, making them potentially cytotoxic [27], were only found among the CD62L^low^ progeny of CD8^+^ T cells co-transferred with CD4^+^ T cells. Importantly, these expression profiles differ from those of resting memory T cells recovered after antigen immunization, which do not co-express perforin and both granzymes and therefore are devoid of killing capacity in the absence of antigen re-stimulation [28]. These “helped” CD8 T cells resemble the fully differentiated cells generated after multiple antigen boosts that kill target cells more efficiently than memory cells or effector CD8^+^ T cells recovered at the peak of the primary response [28]. Overall, these results demonstrated that CD4-help induces the accumulation of fully differentiated CD8^+^CD62L^low^ T_EM_ cells.

### Timing of CD4^+^ T cell help

**Figure 3:**
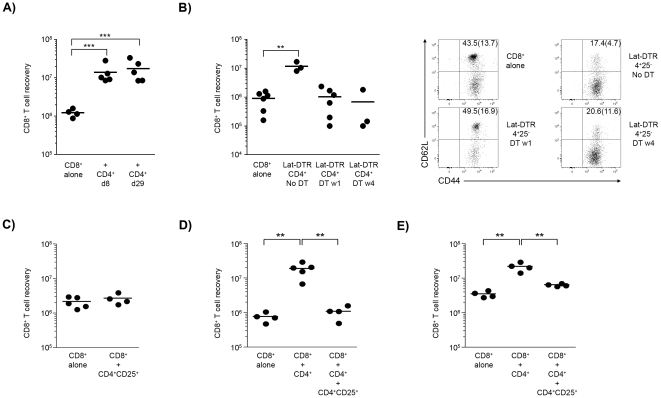
Time requirements of T cell help and the role of T_regs_ cells. (**A**) 2×10^4^ CD8^+^ T cells were transferred into CD3ε^−/−^ hosts. At 8 or 29 days after groups of hosts received CD4 T cells (2×10^4^ cells) and the mice were sacrificed 7 weeks after the first cell transfer. The mean CD8^+^ T cell recovery is shown for the three groups of hosts (***p≤0.001). It should be pointed out that the late transfer of CD4 T cells still induced the preferential accumulation of CD62L^low^CD8^+^ T_EM_ cells (not shown) (**B**) 2×10^4^ CD8^+^ T cells were transferred alone or with CD4^+^ T cells from Lat-DTR mice into a CD3ε^−/−^ host. A fraction of the mice were treated with diphtheria toxin (DT) for 10 days at either one or 4 weeks after cell transfer, and the mice were analyzed 7 weeks after cell transfer. The number of CD8^+^ T cells recovered 7 weeks after cell transfer is shown. CD4^+^ T cell depletion at week 1 or at week 4 reduced the number of CD8^+^ T cells recovered (**p≤0.01). Similar findings were obtained in two independent experiments. Dot plots show representative examples of the CD62L and CD44 expression by the CD8^+^ T cells recovered in the different groups of mice. (**C**) 10^4^ CD8^+^ T cells were transferred into CD3ε^−/−^ hosts alone or with 5×10^4^ CD4 T_reg_ cells. The number of CD8^+^ T cells recovered in individual mice is given as the mean value for each group. (**D**) 10^4^ CD8^+^ T cells were transferred into CD3ε^−/−^ immune-deficient hosts alone, co-transferred with 10^4^ CD4^naive^ T cells or co-transferred with 10^4^ CD4^naive^ T cells and 5×10^4^ CD4 T_reg_ cells in three independent groups of hosts. The number of CD8^+^ T cells recovered in individual mice is given as the mean value for each group. The increased expansion of CD8^+^ T cells seen upon co-transfer with CD4^naive^ T cells was abolished when the T_reg_ cells were added in the transferred mix. (**p≤0.01) (**E**) 10^5^ total CD8^+^ T cells were transferred into CD3ε^−/−^ hosts alone, co-transferred with 10^4^ CD4^naive^ T cells or co-transferred with 10^4^ CD4 T cells and then given 5×10^4^ T_reg_ cells four weeks latter. Mice were culled 8 weeks after the initial CD8^+^ T cell transfer. The data indicate the number of CD8^+^ T cells recovered in each host. Statistically significant differences are shown (**p<0.01). Similar findings were obtained in two independent experiments.

To investigate the timing of CD4-help, CD4^+^ T cells were injected at different times after CD8^+^ T cell transfer. The CD4^+^ T cells enhanced CD8^+^ T cell expansion and differentiation even when transferred one month later (Figure 3A, not shown), indicating that help still occurs in resident populations. Of note, CD4^+^ T cells recoveries were similar in both groups of mice (Figure S2). We also studied whether help required the continuous presence of CD4^+^ T cells using CD4^+^ T cells from mice expressing a human diphtheria toxin receptor (DTR) under the control of the *Lat* gene [29]. In these B6.*Lat* ^fl-dtr^-mice, the administration of DT allowed the selective ablation of CD4^+^ T cells (Figure S2) [23]. Elimination of the Lat-DTR CD4^+^ T cells one week after transfer abrogated the help activity: the number of CD8 T cells recovered was reduced and the majority retained a T_CM_ phenotype (Fig. 3B). Help was also abrogated when the Lat-DTR CD4^+^ T cells were removed 4 weeks after transfer (Figure 3B). However, in this case, T_EM_ were present, but their number was considerably reduced indicating that in the absence of help they failed to survive and decayed after their initial expansion and differentiation in the presence of CD4^+^ T cells (Fig. 3B). These results indicate that CD4-help may induce CD8^+^ T cell expansion at any point in peripheral reconstitution. At the same time, the continuous presence of CD4^+^ T cells appears to be fundamental to promote further CD8^+^ T cell expansion and to ensure the survival and accumulation of CD8^+^T_EM_ cells [30].

Next we determined if help was dependent on the activation and expansion of the donor CD4 T cells. We co-transferred an excess of CD25^+^ T_reg_ cells under conditions that suppress CD4^naïve^ T cell division and accumulation [24]. Under the conditions used in the present study, the transfer of T_reg_ cells did not modify the expansion of the CD8^+^ T cells (Figure 3C) ruling out a direct effect on CD8^+^ T cell expansion. In contrast, by adding an excess of T_reg_ cells in CD8^+^/CD4^naive^ co-transfers we completely abrogated both the expansion of the CD4^+^ T cells (Figure S2) and their helper effect (Figure 3D): the number of CD8 T cells recovered was reduced and the majority retained a T_CM_ phenotype (not shown). More importantly, we found that the transfer of T_reg_ cells one month later still reduced both the number of CD4^+^ T cells (Figure S2) and the CD8^+^ T cell recovery (Figure 3E) mimicking the results observed upon the late administration of DT in hosts with Lat-DTR CD4^+^ T cells. Thus, the increased CD8^+^ T cell accumulation was mediated by the activation and expansion of the co-transferred CD4^naive^ T cells, which could be interrupted at any time point by an excess of T_reg_ cells. Elimination of help prevented survival and accumulation of CD8^+^T_EM_ cells. By comparing CD4^+^ and CD8^+^ T cell recoveries in the individual mice used in these different experiments we found a strong positive correlation (y = 1.16x−29253; p<2.6×10^−17^) between the two T cell subsets (Figure S2). In conclusion, these observations established a quantitative aspect for the helper effect: the CD8^+^ T cell recoveries were proportional to the number of CD4^+^ T cells recovered.

### CD4^+^ T cell help requires CD40 expression by host APCs

**Figure 4:**
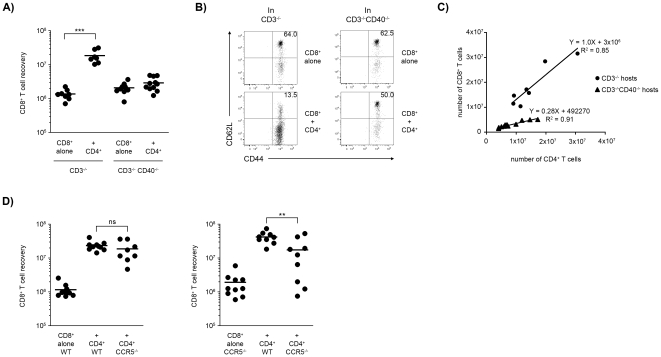
Role of CD40-CD40L interactions and CCR5. (**A**) 2×10^4^ CD8^+^ T cells were transferred alone or co-transferred with 2×10^4^ CD4^+^ T cells into either CD3ε^−/−^ or CD40^−/−^CD3ε^−/−^ mice. The CD8^+^ T cell recovery 8 weeks after transfer is shown from 2-pooled experiments (N = 9). The helper effect observed in CD3ε^−/−^ hosts was absent in CD40^−/−^CD3ε^−/−^ hosts (***p<0.001). (**B**) Dot plots showing representative examples of the CD62L and CD44 expression by CD8^+^ T cells recovered in the different groups of mice. (**C**) Correlation of the number of CD8^+^ and CD4^+^ T cells recovered in individual WT and CD40-deficient hosts. The correlation coefficients are shown (p<0.02 and p<0.001 respectively). Note the different slopes between WT and CD40-deficient hosts. (**D**) 10^4^ CD8^+^ T cells from WT (left) or CCR5^−/−^ (right) were transferred alone or with CD4 T cells from WT or CCR5^−/−^ into CD3ε^−/−^ host. The absolute number of CD8^+^ T cells recovered in the six groups 7 weeks after transfer, pooled from 2 independent experiments. Note that a similar 20-fold enhanced expansion of the WT and CCR5^−/−^ CD8^+^ T cells was detected in the presence of WT CD4^+^ T cells. When both CD8^+^ and CD4^+^ T cells lacked CCR5 from the nine mice studied only two mice showed accumulation of CD8 as that observed in the presence of WT CD4^+^ T cells, and three mice had a lack of CD4-help. The results indicate that to achieve an optimal helper effect the CCR5 receptor should be expressed by either the CD8^+^ or CD4^+^ T cells. Relevant statistically significant differences are shown (**p≤0.01).

We investigated the putative role of CD40 in the CD4^+^ T cell helper effect observed during reconstitution because CD40 deficiencies have been shown to impair memory CD8^+^ T cell responses by interfering in CD4^+^/CD8^+^ T cell collaboration [21], [31], [32]. When transferred into wild-type (WT) CD3ε^−/−^ hosts, CD40^−/−^CD8^+^ T cells responded to CD4^+^ T cell help as well as WT CD8^+^ T cells (Figure S3A). In contrast, when the host was CD40-deficient the WT CD4^+^ T cell helper effect was completely abolished: WT CD8^+^ T cell recovery was low and the majority of the cells remained CD62L^high^ (Figure 4A–B). The absence of help could be due to an inability of donor CD4^+^ T cells to expand in the CD3ε^−/−^CD40^−/−^ host. However, although CD4^+^ T cell expansion was reduced two-fold, the CD4^+^ T cells expanded approximately 30-fold (from 2×10^4^ to >5×10^6^) in CD3ε^−/−^CD40^−/−^ hosts (Figure S3B). Indeed, for similar recoveries of CD4^+^ T cells the number of CD8^+^ T cells recovered was much lower in CD40^−/−^ than in WT hosts (Figure 4C). The slopes of the CD4/CD8 cell number correlations confirm that the CD4 helper effect was faulty in the CD40-deficient hosts (Figure 4C). These findings indicated that CD4^+^ T cell help to CD8^+^ T_EM_ cells required CD40 expression by host cells and introduce a qualitative aspect to the CD4-helper effect; interaction with CD40 was required for CD4^+^ T cell differentiation into full helper functions.

We attempted to identify the CD40^+^ host cell population involved in this response. Because the magnitude of the helper effect and the phenotypic changes induced by CD4^+^ T cells on the CD8^+^ T cells were identical in Rag2^−/−^ and CD3ε^−/−^ hosts (Figure S4) we excluded a critical role of B cells. Therefore the CD4^+^ T cell of CD8^+^ T cells likely required CD40/CD40L interactions with host antigen presenting cells (APCs). We found that the CD4^+^ T cell transfer induced major modifications in the host CD11c^+^APCs. The host CD11c^+^APC CD40 expression was up regulated and the number of these cells increased 3 to 5-fold (Figure S5A). These cells also had a higher frequency of EdU^+^ cells (Figure S5B). These findings suggest that CD11c^+^DCs may act as mediators of the CD4-mediated helper effect on CD8^+^ T cells.

During immune responses, CD4/APC interactions may lead to the secretion of M1P-chemokines that attract CD8^+^ T cells [33]. We investigated whether disruption of the chemokine receptor CCR5 affects CD4-help. We transferred WT and CCR5^−/−^ CD8^+^ T cells either alone or together, with CD4^+^ T cells from WT or CCR5^−/−^ donors. When transferred alone, WT and CCR5-deficient CD8^+^ T cells expanded to similar levels, though expansion seemed increased and more variable in the CCR5-deficient cells (Figure 4D). The WT CD8^+^ T cells expanded 20-fold more in the presence of WT or CCR5-deficient helper T cells though the help was less consistent when CCR5^−/−^CD4^+^ T cells were co-transferred (Figure 4D). Surprisingly, WT CD4^+^ T cells mediated a similar 20-fold increased expansion of the CCR5-deficient CD8^+^ T cells (Figure 4D). However, when both the CD8^+^ and CD4^+^ T cells lacked CCR5, the helper effect was highly variable. Thus, among the nine mice studied the CD8^+^ T cell expansion reached values similar to those observed in the presence of WT CD4^+^ T cells in only two mice, whereas CD4^+^ T cell help was completely absent in three mice (Figure 4D). These findings suggest that optimal helper effects require the expression of a functional CCR5 by both CD8^+^ and CD4^+^ T cells. In the absence of the CCR5 receptor, CD4/CD8 cell encounters could still occur, but instead of being oriented and consistent they would be less frequent and random creating great variability in the helper effects observed.

### Role of type I IFNs and IL-6

**Figure 5:**
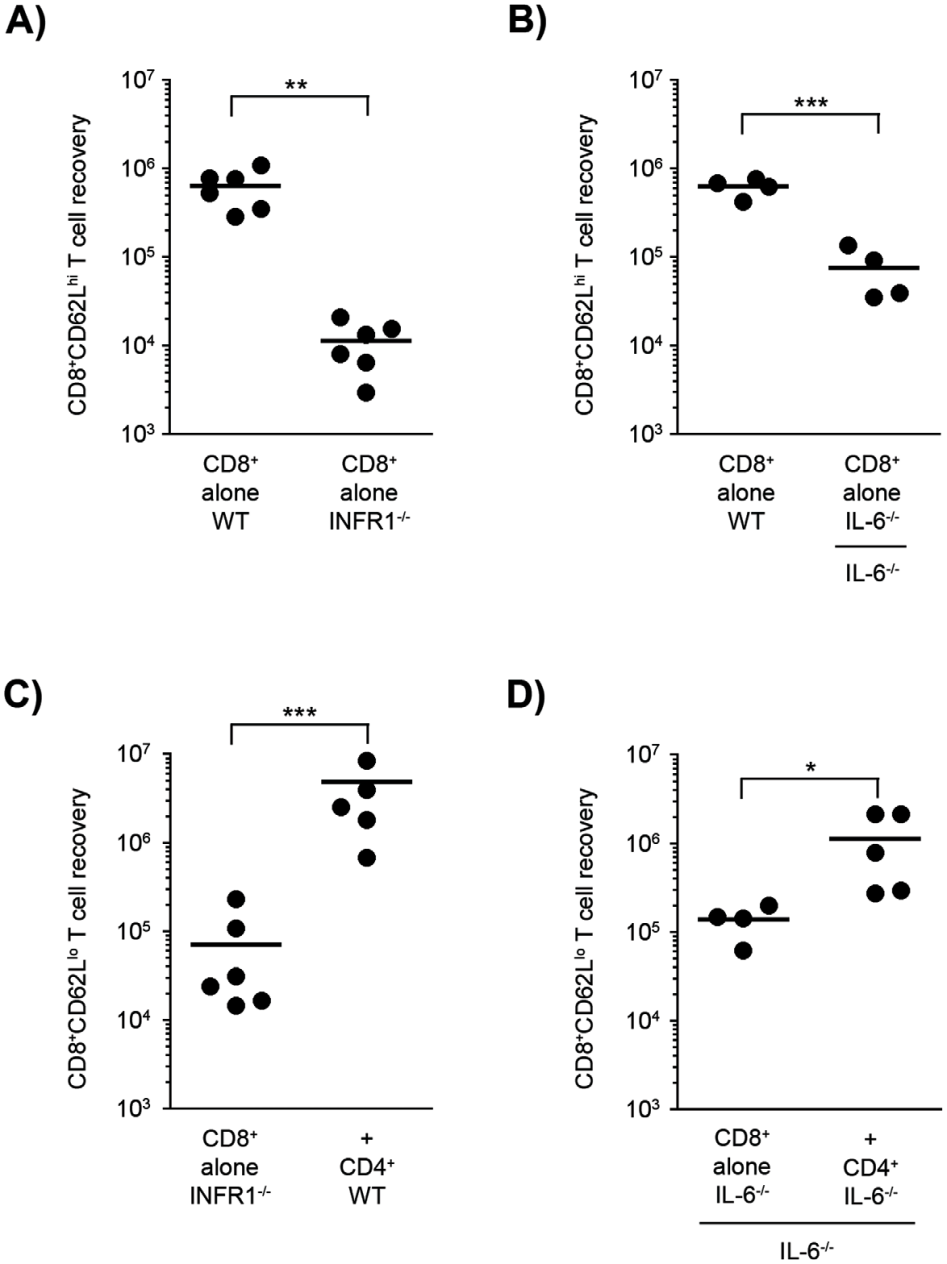
Type I IFN and IL-6 contribute to CD8^+^ T_CM_ cell recovery. (**A**) 2×10^4^ CD8^+^ T cells from WT or IFNAR1^−/−^ mice were transferred into RAG^−/−^ mice. The CD8^+^CD62L^high^ T_CM_ cell recovery after 7 weeks is shown from two pooled experiments. (**B**) The CD8^+^CD62L^high^ T_CM_ cell recovery 8 weeks after the transfer of 2×10^4^ CD8^+^ T cells into RAG^−/−^ or RAG^−/−^IL-6^−/−^ T cell-deficient hosts. (**C**) 2×10^4^ CD8^+^ T cells from WT or IFNAR1^−/−^ mice were transferred alone or with CD4^+^ T cells into RAG^−/−^ mice. The CD8^+^CD62L^low^ T cell recovery after 7 weeks is shown for two pooled experiments. The absence of IFNAR1 expression doesn't prevent the CD8^+^ T cells to receive CD4-help. (**D**) The CD8^+^CD62L^low^ T cell recovery 8 weeks after 2×10^4^ CD8^+^ T cells from IL-6^−/−^ mice were transferred alone or with CD4^+^ T cells into RAG^−/−^IL-6^−/−^ mice. Statistically significant differences are shown (*p≤0.05; **p<0.01; ***p<0.001).

The differential effects of CD4-help suggest that the recoveries of T_CM_ and T_EM_ CD8^+^ T cell subtypes obey different rules. Environmental cytokines induced upon the transfer of cells into immune-deficient hosts could play a role in shaping the helper effect and the recovery of the CD8^+^ T cell subtypes. Among these cytokines, type I IFN and IL-6 are likely candidates as the number of CD8^+^ T cells is reduced in type I IFN- and IL-6-deficient mice [34], [35]. To test the role of these cytokines we used IFNAR^−/−^CD8^+^ T cells, which are unresponsive to Type I IFNs, and IL-6^−/−^ host and donor mice. It should be pointed out that in both cases input CD8 T cell populations, i.e. the frequency of naïve vs. memory CD8 T cells was identical between WT and KO populations (Figure S6). In the absence of type I IFNs signals, the recovery of IFNAR^−/−^CD8^+^CD62L^high^ T_CM_ cells was 100-fold lower than that of WT cells (10^4^ vs. 10^6^; Figure 5A). In the absence of IL-6, the recovery of IL6^−/−^CD8^+^CD62L^high^ T cells was ten-fold lower than that of WT cells (10^5^ vs. 10^6^; Figure 5B). In both cases, the recovery of CD8^+^CD62L^low^ T_EM_ cells was only partial and not significantly reduced (data not shown). Importantly, WT CD4^+^ T cells induced a 100-fold greater expansion of the IFNAR^−/−^CD8^+^CD62L^low^ T cells (i.e. WT CD8^+^ T cell levels). The absence of IL-6 also did not prevent an increased expansion of CD8^+^CD62L^low^ cells in the presence of CD4^+^ T cells (Figure 5D), but recoveries were lower, suggesting that IL-6 may also play a role in CD8^+^CD62L^low^ T cell accumulation. It should be pointed out that in the absence of IL-6 the CD8/CD4 cell number correlation coefficient was y(CD8) = 0,87x(CD4)+183231, less than in WT conditions (y(CD8) = 1.16x(CD4)−29253) (Figure S2). In conclusion, though the recovery of CD8^+^ T_CM_ cells required type I IFN and IL-6, the increased accumulation of CD8^+^ T_EM_ cells induced by CD4-help was largely independent of type I IFN while IL-6 partially contributes to the CD4 helper effects.

### Role of IL-2 and IL-15

**Figure 6:**
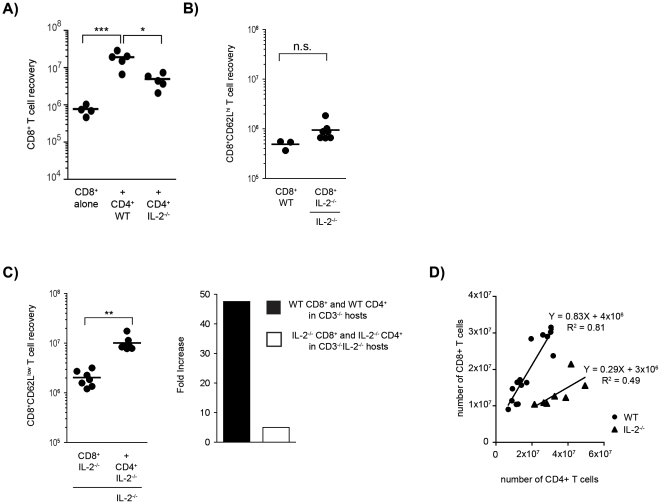
Role of IL-2 in the CD4^+^-dependent accumulation of CD8^+^ T cells. (**A**) 1.5×10^4^ CD8^+^ T cells from WT mice were transferred alone or with equal numbers of CD4^naive^ T cells from WT or IL-2-deficient mice into CD3ε^−/−^ hosts. CD8^+^ T cell recovery in individual hosts of the 3 groups is shown. (**B**) 2×10^4^ CD8^+^ T cells from IL-2-deficient mice were transferred alone or with CD4 T cells into immune-deficient WT or IL-2^−/−^ hosts. The CD8^+^CD62L^high^ T_CM_ cell recovery after 7 weeks is shown for two pooled experiments. (**C**) The left panel shows the CD8^+^CD62L^low^T_EM_ cell recovery after 7 weeks in IL-2^−/−^ hosts from two pooled experiments. The right panel shows the fold increases of CD8^+^ T_EM_ cell recovery calculated by dividing the number of CD8^+^ T cells recovered in the presence of CD4^+^ T cells by the number of CD8^+^ T cells recovered in the absence of CD4^+^ T cells. CD8^+^CD62L^low^ T cell recovery increased about 50-fold, under WT conditions, but only 5-fold in the absence of IL-2. (**p≤0.01). WT data pooled from 3 different experiments (**D**) Correlation of the number CD8^+^ T cells and CD4^+^ T cells recovered in individual hosts in the presence or in the absence of IL-2. The correlation coefficients are shown (p<0.0001 and p<0.03 respectively). Data pooled from 3 different experiments.

The expansion of CD4^+^ T cells in lymphopenic hosts is accompanied by the production of IL-2, which is crucial in the regulation of CD4^+^CD25^+^ T_reg_ cell homeostasis [14], [24], anti-tumor CD8^+^ T cell responses [36] and the generation of CD8^+^ T cell memory [37]. We tested the role of IL-2 in the recovery of CD8^+^ T cell subsets. We found that when IL2^−/−^ CD4^+^ T cells were used as helpers in an IL-2 sufficient environment they were able to expand, but their help was significantly reduced, although not absent (figure 6A). We concluded that CD4-derived IL-2 is required for optimal help but that other IL-2 sources may also be involved. Thus, we tested the helper effect in complete absence of IL-2 by transferring IL-2^−/−^CD4^+^ T cells and IL-2^−/−^CD8^+^ T cells into IL2^−/−^CD3ε^−/−^ hosts. We found that when the host and CD8^+^ cells were IL-2-deficient, the recovery of CD8^+^CD62L^high^ cells was not altered (Figure 6B). In contrast, in total absence of IL-2 the helper effect was reduced, but not abrogated (Figure 6C). The study of CD8/CD4 number correlations confirmed that the CD4 helper effect was defective in the absence of IL-2 (Figure 6D): for similar recoveries of CD4^+^ T cells the number of CD8^+^ T cells recovered was much lower without IL-2. We concluded that IL-2 produced or induced by the helper CD4^+^ T cells plays a role in the increased accumulation of the CD8^+^ T_EM_ cells but since help was still observed in its absence other mechanisms may also play a role.

**Figure 7:**
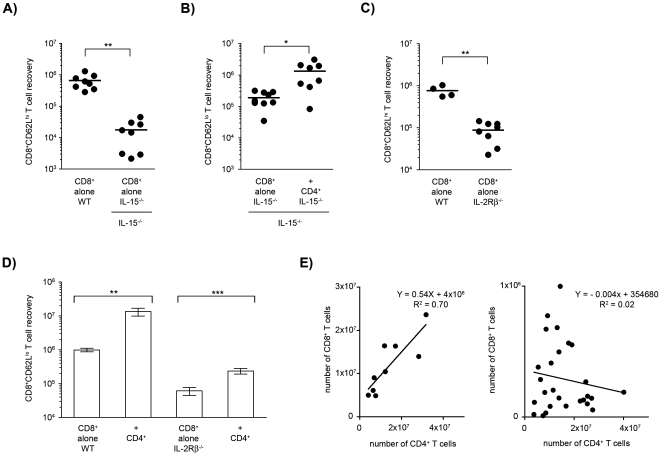
Role of IL-15 in the CD4^+^-dependent accumulation of CD8^+^ T cells. (**A**) The number of CD8^+^CD62L^high^ T cells recovered in Rag^−/−^ and in Rag^−/−^IL-15^−/−^ hosts after 2×10^4^ naïve CD8^+^ T cells from WT or IL-15^−/−^ mice were transferred alone or with CD4^+^ T cells into immune-deficient WT or IL-15^−/−^ hosts. (**B**) 2×10^4^ naïve CD8^+^ T cells from IL-15^−/−^ mice were transferred alone or with IL-15^−/−^ CD4^+^ T cells into immune-deficient IL-15^−/−^ hosts. The CD8^+^CD62L^low^ T cell recovery after 4 weeks is shown for two pooled experiments. In the complete absence of IL-15, we observed an increased accumulation of the CD8^+^ (CD62L^low^) T cells in presence of the CD4^+^ T cells. Statistically significant differences are shown (*p≤0.05; **p<0.01). (**C**) The CD8^+^CD62L^high^ T_CM_ cell recovery 7 weeks after transferring 2×10^4^ naïve CD8^+^ T cells from WT or IL-2Rβ^−/−^ donors alone into immune-deficient mice. Similar results were obtained in three independent experiments (**p≤0.01). (**D**) 2×10^4^ naïve CD8^+^ T cells from WT (left) or IL-2Rβ^−/−^ donors (right) were injected alone or with 2×10^4^ CD4^+^ T cells into immune-deficient hosts. The bars indicate the mean±se of CD8^+^ T_EM_ cell recovery after 8–9 weeks from four pooled experiments (n = 20–24) (**p≤0.01; ***p<0.001). (**E**) Correlation of the number of WT (left) or IL-2Rβ^−/−^ (right) CD8^+^ T cells and WT CD4^+^ T cells recovered in individual hosts. The correlation coefficients are shown (p<0.0001 and NS respectively).

As it has been shown that IL-15 is an important cytokine for CD8^+^ T cell homeostasis, we studied CD8^+^ T cell recoveries and the CD4-mediated helper effect in the absence of IL-15. Upon transfer of IL-15^−/−^CD8^+^ T cells into IL-15^−/−^ lymphopenic hosts, CD8^+^CD62L^high^ T cells recovery was poor (Figure 7A), but IL-15^−/−^CD4^+^ T cells were still able to enhance the accumulation of IL-15^−/−^CD8^+^CD62L^low^ T cells (Figure 7B). The IL-15^−/−^CD8^+^CD62L^low^ T cell recovery was, however, less than that observed under IL-15 sufficient conditions and the CD8/CD4 correlation coefficient (y(CD8) = 0,47x(CD4)–19237) lower than in WT conditions, but higher than in absence of IL-2. We concluded that IL-15 plays an important role in CD8^+^CD62L^high^ T cell survival/accumulation while it also contributes to the CD4-mediated helper effect that induces the CD8^+^CD62L^low^ T cell expansion.

Since the IL-2Rβ-chain is common to signaling mediated by both IL-2 and IL-15, we investigated its requirement in CD8^+^ T cell expansion. The recovery of CD8^+^CD62L^high^ T cells upon transfer of IL-2RβCD8^+^ T cells was consistently poor (Figure 7C). Importantly, the recovery of IL-2RβCD62L^low^ T_EM_ cells was also reduced. In the presence of CD4^+^ T cells their increase was reduced and more importantly, independent of the number of CD4^+^ T cells present (Figure 7D, Figure S7), suggesting that they responded to factors produced by the host as a consequence of the environmental changes induced. We concluded that the signals mediated by IL-2Rβ strongly affect the accumulation of both CD8^+^CD62L^high^ and also of the CD8^+^CD62L^low^ T cells accumulating as the result of IL-2- and IL-15-mediated CD4 helper effects.

## Discussion

In the present study, we compared the recoveries of CD44^+^CD62L^high^(T_CM_) and CD44^+^CD62L^low^(T_EM_) CD8^+^ T cells in different experimental settings and the absence or presence of co-transferred CD4^+^ T cells. On the whole, our findings indicate that the accumulation of T_CM_ and T_EM_ cells is differentially regulated during the reconstitution of the peripheral T cell pools of immune-deficient hosts. Therefore, though most of CD8^+^ T cells recovered after the transfer of CD8^+^ T cells alone were CD62L^high^, the presence of CD4^+^ T cells promoted a massive increase in the accumulation of CD8^+^CD62L^low^ cells, which are fully differentiated and express more killer function molecules than memory cells or effector CD8 cells recovered at the peak of the primary immune response [28]. Why such fully effector CD8^+^ T_EM_ cells are generated during peripheral T cell reconstitution is unclear. We may assume that the differentiation to effector functions represents a priority during immune reconstitution to ensure protective functions [19]. However, fast differentiation of effector CD8^+^ T cells and the recovery of protective functions seems to require CD4-cell help, as only in the presence of CD4^+^ T cells can CD8^+^ T cells express an increased frequency of effector molecule mRNAs or generate some type of protective memory after LDP [20], [38]. If unchecked, this strong helper effect may lead to a massive accumulation of fully differentiated CD8^+^ T cells, which may be protective or deleterious according to the environmental context. These observations are of particular relevance in therapeutic situations in which reconstitution of the peripheral T cell pools demands the rapid re-establishment of CD8^+^ T cell effector functions.

Understanding the cellular interactions that promote the help effect is essential to being able to manipulate peripheral reconstitution. The CD4^+^ T cell help effect was immediate, as it was observed early after transfer and persisted with time: it acted on both recently introduced and resident CD8^+^ T cells. More importantly, the effect required the continuous presence of CD4^+^ T cells, suggesting that first it induces an increased proliferation of CD8^+^ T_EM_ cells and later ensures their survival and accumulation. Indeed, deletion of *Lat* ^fl-dtr^CD4^+^ T cells either 1 week or 4 weeks after transfer resulted in a marked decline in the number of CD8^+^ T_EM_ cells recovered. Moreover, even at late times after the transfer, the frequency of proliferating CD8^+^ T cells was higher in the presence of helper cells, suggesting that a continuing interaction between the two cells is required to ensure CD8^+^ T cell survival. Thus, our findings first establish a quantitative feature for help by showing a strong positive correlation between the numbers of CD8^+^ and CD4^+^ T cells; the higher the expansion of CD4^+^ T cells the higher were the CD8^+^ T cell recoveries. Once the CD4-expansion was abolished and the CD4 numbers reduced the CD8^+^ T_EM_ cells failed to survive and their accumulation lessened. Indeed, CD4-help could be abrogated by the simultaneous or late transfer of T_reg_ cells, which suppress CD4^naïve^ T cell expansion [24]. Notably, T_reg_ cells have been reported to suppress CD8^+^ T cell immune responses *in vitro* [39] and *in vivo* [36], [40], [41], but their role in CD8^+^ T cell reconstitution was still unclear. Here, T_reg_ cells alone did not modify CD8^+^ T cell recovery, indicating that T_reg_ cells can regulate CD8^+^ T cell LDP by down modulating CD4^+^ T cell activation [36]. Thus, protocols that deplete T_reg_ cells may favor CD8^+^ T cell responses [40], [41] also by boosting CD4^+^ help rather than simply preventing their direct effect in CD8^+^ T cells. The T_regs_ can therefore, be used to abridge the self-aggressive behavior of the fully differentiated CD8^+^ T_EM_ cells resulting from a deregulated CD4 T cell response. Damping the inflammatory response induced by CD4^+^ T cells with T_reg_ cells abrogates help and may also prevent CD8-mediated self-aggression.

We gathered strong evidence indicating that CD4^+^ T cell help of CD8^+^ T cells requires the intervention of third party host APCs, and that this effect is CD40-dependent [20]. In response to a lymphopenic environment, donor CD4^+^ T cells induce the up-regulation of CD40 and the activation of host APCs, creating a positive feedback loop that further sustains CD4^+^ T cell expansion. When CD40 was absent from the host environment the strong positive correlation between CD8 and CD4 T cell numbers was lost. The lack of CD4 T cell help in the CD40-deficient hosts, despite their >50-fold expansion, introduces a new qualitative facet to CD4 help; it suggests that CD4^+^ T cells require interactions with CD40 and/or co-stimulation molecules in the host APCs [42] in order to differentiate and be fully licensed to mediate helper effects. In view of our current findings it is likely that the in vivo use of CD40-agonists [43], [44] may ensure fast effector cell recoveries during lymphopenia restoration.

The help effect likely requires the formation of APC/CD4/CD8 cell clusters. During immune responses, interactions between CD4 cells and APCs have been shown to lead to the secretion of CCL3 and CCL4 chemokines that are supposed to attract CCR5^+^CD8^+^ T cells to the APC/CD4^+^ T cell clusters [45]. We showed that CCR5 expression by CD8^+^ T cells is not required for the helper effects observed during reconstitution. However, optimal helper effects required the expression of a functional CCR5 chemokine receptor by at least one of the two intervening populations, suggesting that both activated CD4^+^ and CD8^+^ T cells secrete chemokines that attract opposite CCR5^+^ partners [46]. In the absence of CCR5 a cell subset would be unable to respond to the chemokine, but it could still attract other CCR5^+^ subsets. In contrast, when both cell populations lack CCR5, CD4/CD8 T cell encounters would occur purely at random and, thus, less frequently and less efficiently, resulting in a wide-range of variation in helper effects. Because the CCL3 and CCL4 chemokines are produced mainly at sites of APC-T cell interactions [33], our findings would suggest that CD4/CD8 T cell interactions likely occur in close vicinity to host APCs. The results also indicate that activated CD8^+^ T cells are able to maintain a chemokine gradient that enhances the establishment of interacting cell clusters.

Interestingly, though CD4-help in both LDP and conventional responses require third party APCs and a close vicinity of the three intervening populations [33], the CD8^+^ T_EM_ cells generated during LDP or conventional responses differ in CD40 requirements. We showed that, during LDP, the CD4-help of CD8^+^ T cells was dependent on CD40^+^APCs, whereas CD8^+^ T cells might also receive CD4-help directly through CD40 during conventional responses [21]. Because CD8 reconstitution requires sustained help from licensed CD4^+^ T cells despite transient help sufficing to generate memory CD8^+^ T cells during immune responses [21], we postulate that, during a response, the presence of high loads of antigen allow stimulation of the specific CD8 T cells by helper cells [21] whereas, during LDP, low loads of antigen specific for each of the responding CD8 T cells require additional stimulation by the host CD40^+^APCs.

The different impact of CD4^+^ T cell-dependent help on the different CD8^+^ T cell subsets prompted us to investigate the signals involved in T_CM_ and T_EM_ CD8^+^ T cell repopulation. Reconstitution of the peripheral CD8^+^ T cell pool, besides requiring TCR-MHC recognition is generally believed to be mostly dependent of IL-15 and IL-7 mediated signals [4], [5], [47]. We confirmed the role of IL-15 and IL-2Rβ-mediated signals, but further identified a major role of IL-6 and type I IFNs in CD8^+^ T_CM_ cell expansion. The absence of IL-6 had a similar impact as IL-15 deprivation reducing CD8^+^ T_CM_ cell yields by 10-fold. Surprisingly, the effect of type I IFNs was greater because IFNAR^−/−^ CD8^+^ T_CM_ cell yields were reduced 100-fold compared to WT cells. In light of these findings, lower Type I IFNs and IL-6 levels likely contribute to the observation that germ-free immune-deficient hosts do not support the marked T cell LDP observed in conventionally raised mice [48]. Interestingly, in adult mice the frequency of naïve vs. memory CD8 T cells is identical between IFNAR^−/−^, IL-6^−/−^ and WT populations suggesting that the role of these cytokines is only determinant during CD8 T cell responses. Indeed, type I IFNs and IL-6 have been shown to be required in the control of viral infections, [34], [35] and type I IFNs play a role in the generation of effector and memory CD8^+^ T cells [34]. Here we described a mechanism through which type I IFNs and IL-6 could promote efficient secondary immune responses by facilitating the accumulation of CD8^+^ T_CM_ cells. In contrast, the accumulation of CD8^+^ T_EM_ cells was completely independent of type I IFNs, while IL-6 and IL-15 participate in their accumulation in presence of CD4 help. IL-6 has been shown to be involved in CD8-mediated colitis arising during lymphopenia [49] in agreement with our data showing a role for IL-6 in T_EM_ accumulation.

IL-2 and IL-15 signals using the IL-2Rβ chain expressed by the CD8^+^ T cells, mediated mostly the CD8^+^ T_EM_ cell recovery. Indeed, CD4-help of CD8^+^ T_EM_ cells was reduced in the absence of IL-2, IL-15 or when the CD8^+^ T cells were IL-2Rβ-deficient. CD8^+^ T cell expansion has been reported to occur when these cells are exposed to increased IL-2 levels [50] or during adoptive cancer immunotherapy using antigen-specific CD8^+^ T cells in the presence of IL-2-sufficient CD4^+^ T cells [36]. We confirmed that CD4-derived IL-2 plays a role in the selective expansion of CD8^+^ T_EM_ cells during peripheral reconstitution of lymphopenic hosts, but we have also gathered evidence supporting a role for IL-15. Comparing directly the effects of the absence of IL-2 or IL-15 we found that while the absence of IL-15 impinges strongly in the T_CM_ cell recovery and less in T_EM_ cell recovery, the absence of IL-2 does not alter T_CM_ cell recovery and affects exclusively T_EM_ cell recovery. The observed increased accumulation of IL-2Rβ-deficient T_EM_ cells was independent of the number of CD4^+^ T cells recovered, suggesting that they are likely expanding in response to environmental inflammation induced by the activated helper cells. The recent report that CD8^+^ T cells expand extensively once transferred into IL-2Rβ-deficient mice despite the presence of increased numbers of activated CD8^+^ T cells [50], suggests that two CD8^+^ T cell types occupy different niches and probably belong to different kin: one dependent on IL-2/IL-15 and the other, present in the IL-2Rβ-deficient hosts, not. IL-2-deficient mice also show uncontrolled CD4^+^ T cell activation and increased number of “IL-2 independent” CD8^+^ T cells. [51] The CD8^+^ T cells from IL-2Rβ-deficient mice are selected in absence of IL-2 and IL-15 signals and therefore likely to be more reactive to other cytokines. We suggest that IL-6 is a likely candidate, but we cannot rule out the existence of other yet unidentified factors able to promote CD8^+^ T_EM_ cell expansion during this LDP helper response.

Our findings uncover novel aspects of the interactions between co-expanding T cells in response to a lymphopenic state, which may be particularly relevant to clinical attempts at reconstituting the peripheral T cell pool or boosting tumor-specific immune responses. The expansion of CD4^+^ T cells leads to the expansion of a differentiated CD8^+^ T_EM_ cell pool. Importantly, CD8^+^ T_CM_ cell recovery is not significantly altered, pointing to several dichotomies in the survival and expansion of different CD8^+^ T cell subsets during peripheral T cell recovery. Though the maintenance of CD8^+^ T_CM_ cell numbers is strictly dependent on IFNAR expression and strongly requires IL-6 and IL-15, the expansion of CD8^+^ T_EM_ cells does not, as it is mainly dependent on CD4-help to bypass such requirements; while the expansion and maintenance of CD8^+^ T_CM_ cell numbers can occur in the absence of help, the expansion of CD8^+^ T_EM_ is strongly determined by CD4^+^ T cell help. The segregation of the signals controlling the CD8^+^ T cell subsets allows lenient control of fully differentiated CD8^+^ T_EM_ cells, which massively increase once a CD4^+^ T cell “perturbation” is introduced, providing the immune system with the capacity to readily adapt during repeated antigenic challenges to ensure immediate effector responses [52], while the number of CD8^+^ T_CM_ cells remains under control to confer future protection [53]. On the whole our results unveil the complexity of CD4 helper effects to CD8 T cells demonstrating that, during LDP, help is determined by multiple factors that operate differently according to the environmental settings. Among these settings expression of IL-2Rβ by CD8 cells bests CD40 expression by host APCs, and among cytokines there is a hierarchy where IL-2>IL-15>IL-6.

## Supporting Information

Figure S1 **Effects of CD4-help on CD8 T cell division and death rates.** (**A**) CD8^+^ T cells were stained with CFSE and 10^6^ injected alone or with 2×10^4^ CD4^+^ T cells into CD3ε^−/−^ mice. The CFSE staining of the CD8^+^ T cells recovered by day 4 is shown. The percentage of CFSE^+^ and CFSE^−^ cells is shown in the respective gate. Similar results were obtained in a second independent experiment. (**B**) 2×10^4^ CD8^+^ T cells were injected alone or with CD4^+^ T cells into CD3ε^−/−^ mice. After 10 days, host mice were treated with EdU. Three days latter cells were stained for EdU and Annexin V expression. The upper histograms show EdU staining among the LN CD8^+^ T cells for one representative host (out of 5). The fraction of Edu stained cells is shown. The lower histograms show the Annexin V staining among the LN CD8^+^ T cells for one representative host (out of 3). The fraction of Annexin V stained cells is shown. Similar results were obtained in a second independent experiment. (**C**) The histograms show the CCR7 expression among the recovered CD8^+^ T cells. The MFI is provided. CCR7 expression by CD8^+^ T cells was reduced in presence of the CD4^+^ T cells (dotted line).
(TIF)

Figure S2 **CD4^+^ T cell recoveries.** (**A**) The absolute number of CD4^+^ T cells recovered after 2×10^4^ CD4^+^ T cells were transferred 8 or 29 days after the transfer of 2×10^4^ CD8^+^ T cells (corresponding to data in Figure 3A). (**B**) The absolute number of LAT-DTR CD4^+^ T cells recovered 8 weeks after 2×10^4^ LAT-DTR CD4^+^ T cells were transferred together with 2×10^4^ CD8^+^ T cells into host mice that were either left untreated or treated with DT 1 week or 4 weeks after transfer. (**C**) The absolute number of CD4^+^ T cells recovered 8 weeks after 10^4^ CD4^+^ T cells were co-transferred with 2×10^4^ CD8^+^ T cells, co-transferred with 2×10^4^ CD8^+^ T cells and 5×10^4^ T_reg_ cells or co-transferred with 2×10^4^ CD8^+^ T cells into host mice that received 5×10^4^ T_reg_ cells 4 weeks after transfer. (**D**) Correlation of the number of CD8^+^ and CD4^+^ T cells recovered in individual mice from the experiments shown in Figure 3. The correlation coefficients are shown (p<2.6×10^−17^).
(TIF)

Figure S3 **Expansion of CD8^+^CD40^−/−^ T cells.** (**A**) The absolute number of CD8^+^ T cells recovered 8 weeks after 2×10^4^ CD8^+^ T cells from WT or CD40^−/−^ donors were transferred alone or with CD4 T cells into CD3ε^−/−^ mice. The increased accumulation of CD8^+^ T cells was observed regardless of whether these cells were able to express CD40. (**B**) **Control of the growth of the CD4^+^ T cells in CD40^−/−^ hosts.** CD4^+^ T cell recovery 10 weeks after the of the transfer of 2×10^4^ CD4^+^ T cells co-injected with WT or CD40^−/−^ donor T cells into CD3ε^−/−^ or CD40^−/−^CD3ε^−/−^ hosts. Note that CD4^+^ T cell expansion still occurred in the absence of CD40. Results are from two pooled experiments. Statistically significant differences are shown (*p≤0.05; ***p<0.001).
(TIF)

Figure S4 **Help in RAG^−/−^ host mice.** The absolute number of CD8^+^ T cells recovered 7 weeks after 2×10^4^ CD8^+^ T cells were transferred alone or with CD4 T cells into CD3ε^−/−^ or RAG^−/−^ hosts. The helper effect was observed in the presence or absence of B cells. Statistically significant differences are shown (***p<0.001).
(TIF)

Figure S5 **Role of CD11c^+^ APCs.** (**A**) CD40 expression among the host CD11c^+^ cells (left) and the total number of host CD11c^+^ cells (right) recovered 17 days after 2×10^4^ CD8^+^ T cells were transferred alone or with 2×10^4^ CD4^+^ T cells into CD3ε^−/−^ mice. (**p≤0.01). Values outside the histogram represent the mean±se (right). To enumerate CD11c^+^ cells, spleen and LNs were incubated 45 min at 37°C in RPMI containing DNAse I (50 µg/ml) and collagenase type IV (1 mg/ml). In the presence of CD4^+^ T cells, the number of CD11c^+^ cells and their CD40 expression increased. CD4^+^ T cell transfer did not modify CD80, CD86 or MHC class II expression by the host APCs (not shown). (**B**) The EdU staining among CD11c^+^ cells in the spleen for one representative host (out of 5). The fraction of Edu stained cells is shown. Similar results were obtained in two independent experiments.
(TIF)

Figure S6 **Phenotype of CD8^+^ T cells.** Dot plots show the phenotype of donor CD8^+^ T cells from WT (left); INFR1^−/−^ (middle) and IL-6^−/−^ mice.
(TIF)

Figure S7 **Role of IL-2 in CD4^+^ T cell help.** The fold increases of CD8^+^ T cell recovery 8 weeks after the transfer of 2×10^4^ CD8^+^ T cells alone or with 2×10^4^ CD4^+^ T cells into CD3ε^−/−^ mice, calculated by dividing the number of CD8^+^ T cells recovered in the presence of CD4^+^ T cells by the number of CD8^+^ T cells recovered in the absence of CD4^+^ T cells. CD8^+^ T cell recovery increased 10 to 30-fold in the presence of WT CD4^+^ T cells, but only 2 to 3-fold in the total absence of IL-2 or if they lacked the IL-2Rβ chain.
(TIF)

## References

[1] Rocha B, Dautigny N, Pereira P (1989). Peripheral T lymphocytes: expansion potential and homeostatic regulation of pool sizes and CD4/CD8 ratios in vivo.. European Journal of Immunology.

[2] Ernst B, Lee DS, Chang JM, Sprent J, Surh CD (1999). The peptide ligands mediating positive selection in the thymus control T cell survival and homeostatic proliferation in the periphery.. Immunity.

[3] Goldrath AW, Bevan MJ (1999). Low-affinity ligands for the TCR drive proliferation of mature CD8+ T cells in lymphopenic hosts.. Immunity.

[4] Tan JT, Dudl E, LeRoy E, Murray R, Sprent J (2001). IL-7 is critical for homeostatic proliferation and survival of naive T cells.. Proc Natl Acad Sci U S A.

[5] Schluns KS, Kieper WC, Jameson SC, Lefrancois L (2000). Interleukin-7 mediates the homeostasis of naive and memory CD8 T cells in vivo.. Nat Immunol.

[6] Seddon B, Tomlinson P, Zamoyska R (2003). Interleukin 7 and T cell receptor signals regulate homeostasis of CD4 memory cells.. Nat Immunol.

[7] Jameson SC (2002). Maintaining the norm: T-cell homeostasis.. Nat Rev Immunol.

[8] Hao Y, Legrand N, Freitas AA (2006). The clone size of peripheral CD8 T cells is regulated by TCR promiscuity.. J Exp Med.

[9] La Gruta NL, Driel IR, Gleeson PA (2000). Peripheral T cell expansion in lymphopenic mice results in a restricted T cell repertoire.. Eur J Immunol.

[10] Goldrath AW, Luckey CJ, Park R, Benoist C, Mathis D (2004). The molecular program induced in T cells undergoing homeostatic proliferation.. Proc Natl Acad Sci U S A.

[11] Tanchot C, Le Campion A, Martin B, Leaument S, Dautigny N (2002). Conversion of naive T cells to a memory-like phenotype in lymphopenic hosts is not related to a homeostatic mechanism that fills the peripheral naive T cell pool.. J Immunol.

[12] Maloy KJ, Powrie F (2001). Regulatory T cells in the control of immune pathology.. Nat Immunol.

[13] King C, Ilic A, Koelsch K, Sarvetnick N (2004). Homeostatic expansion of T cells during immune insufficiency generates autoimmunity.. Cell.

[14] Almeida AR, Zaragoza B, Freitas AA (2006). Indexation as a novel mechanism of lymphocyte homeostasis: the number of CD4+CD25+ regulatory T cells is indexed to the number of IL-2-producing cells.. J Immunol.

[15] Sallusto F, Lenig D, Forster R, Lipp M, Lanzavecchia A (1999). Two subsets of memory T lymphocytes with distinct homing potentials and effector functions.. Nature.

[16] Sakaguchi S (2004). Naturally arising CD4+ regulatory t cells for immunologic self-tolerance and negative control of immune responses.. Annu Rev Immunol.

[17] Sakaguchi S, Sakaguchi N, Asano M, Itoh M, Toda M (1995). Immunologic self-tolerance maintained by activated T cells expressing IL-2 receptor alpha-chains (CD25). Breakdown of a single mechanism of self-tolerance causes various autoimmune diseases.. Journal of Immunology.

[18] Shevach EM (2000). Regulatory T cells in autoimmmunity.. Annu Rev Immunol.

[19] Freitas AA, Rocha B (2000). Population biology of lymphocytes: the flight for survival.. Annu Rev Immunol.

[20] Hamilton SE, Jameson SC (2008). The nature of the lymphopenic environment dictates protective function of homeostatic-memory CD8+ T cells.. Proc Natl Acad Sci U S A.

[21] Bourgeois C, Rocha B, Tanchot C (2002). A role for CD40 expression on CD8+ T cells in the generation of CD8+ T cell memory.. Science.

[22] Bevan MJ (2004). Helping the CD8(+) T-cell response.. Nat Rev Immunol.

[23] Mingueneau M, Roncagalli R, Gregoire C, Kissenpfennig A, Miazek A (2009). Loss of the LAT adaptor converts antigen-responsive T cells into pathogenic effectors that function independently of the T cell receptor.. Immunity.

[24] Almeida AR, Legrand N, Papiernik M, Freitas AA (2002). Homeostasis of peripheral CD4+ T cells: IL-2R alpha and IL-2 shape a population of regulatory cells that controls CD4+ T cell numbers.. J Immunol.

[25] Peixoto A, Monteiro M, Rocha B, Veiga-Fernandes H (2004). Quantification of multiple gene expression in individual cells.. Genome Res.

[26] Veiga-Fernandes H, Walter U, Bourgeois C, McLean A, Rocha B (2000). Response of naive and memory CD8+ T cells to antigen stimulation in vivo.. Nat Immunol.

[27] Russell JH, Ley TJ (2002). Lymphocyte-mediated cytotoxicity.. Annu Rev Immunol.

[28] Peixoto A, Evaristo C, Munitic I, Monteiro M, Charbit A (2007). CD8 single-cell gene coexpression reveals three different effector types present at distinct phases of the immune response.. J Exp Med.

[29] Helft J, Jacquet A, Joncker NT, Grandjean I, Dorothee G (2008). Antigen-specific T-T interactions regulate CD4 T-cell expansion.. Blood.

[30] Sun JC, Williams MA, Bevan MJ (2004). CD4+ T cells are required for the maintenance, not programming, of memory CD8+ T cells after acute infection.. Nat Immunol.

[31] Noelle RJ (1996). CD40 and its ligand in host defense.. Immunity.

[32] Grewal IS, Flavell RA (1998). CD40 and CD154 in cell-mediated immunity.. Annu Rev Immunol.

[33] Castellino F, Huang AY, Altan-Bonnet G, Stoll S, Scheinecker C (2006). Chemokines enhance immunity by guiding naive CD8+ T cells to sites of CD4+ T cell-dendritic cell interaction.. Nature.

[34] Kolumam GA, Thomas S, Thompson LJ, Sprent J, Murali-Krishna K (2005). Type I interferons act directly on CD8 T cells to allow clonal expansion and memory formation in response to viral infection.. J Exp Med.

[35] Kopf M, Baumann H, Freer G, Freudenberg M, Lamers M (1994). Impaired immune and acute-phase responses in interleukin-6-deficient mice.. Nature.

[36] Antony PA, Piccirillo CA, Akpinarli A, Finkelstein SE, Speiss PJ (2005). CD8+ T cell immunity against a tumor/self-antigen is augmented by CD4+ T helper cells and hindered by naturally occurring T regulatory cells.. J Immunol.

[37] Williams MA, Tyznik AJ, Bevan MJ (2006). Interleukin-2 signals during priming are required for secondary expansion of CD8+ memory T cells.. Nature.

[38] Hamilton SE, Wolkers MC, Schoenberger SP, Jameson SC (2006). The generation of protective memory-like CD8+ T cells during homeostatic proliferation requires CD4+ T cells.. Nat Immunol.

[39] Piccirillo CA, Shevach EM (2001). Cutting edge: control of CD8+ T cell activation by CD4+CD25+ immunoregulatory cells.. J Immunol.

[40] Kursar M, Bonhagen K, Fensterle J, Kohler A, Hurwitz R (2002). Regulatory CD4+CD25+ T cells restrict memory CD8+ T cell responses.. J Exp Med.

[41] Murakami M, Sakamoto A, Bender J, Kappler J, Marrack P (2002). CD25+CD4+ T cells contribute to the control of memory CD8+ T cells.. Proc Natl Acad Sci U S A.

[42] Hagen KA, Moses CT, Drasler EF, Podetz-Pedersen KM, Jameson SC (2004). A role for CD28 in lymphopenia-induced proliferation of CD4 T cells.. J Immunol.

[43] Diehl L, den Boer AT, Schoenberger SP, van der Voort EI, Schumacher TN (1999). CD40 activation in vivo overcomes peptide-induced peripheral cytotoxic T-lymphocyte tolerance and augments anti-tumor vaccine efficacy.. Nat Med.

[44] French RR, Chan HT, Tutt AL, Glennie MJ (1999). CD40 antibody evokes a cytotoxic T-cell response that eradicates lymphoma and bypasses T-cell help.. Nat Med.

[45] Castellino F, Germain RN (2006). Cooperation between CD4+ and CD8+ T cells: when, where, and how.. Annu Rev Immunol.

[46] Cocchi F, DeVico AL, Garzino-Demo A, Arya SK, Gallo RC (1995). Identification of RANTES, MIP-1 alpha, and MIP-1 beta as the major HIV-suppressive factors produced by CD8+ T cells.. Science.

[47] Tan JT, Ernst B, Kieper WC, LeRoy E, Sprent J (2002). Interleukin (IL)-15 and IL-7 Jointly Regulate Homeostatic Proliferation of Memory Phenotype CD8(+) Cells but Are Not Required for Memory Phenotype CD4(+) Cells.. J Exp Med.

[48] Kieper WC, Troy A, Burghardt JT, Ramsey C, Lee JY (2005). Recent immune status determines the source of antigens that drive homeostatic T cell expansion.. J Immunol.

[49] Tajima M, Wakita D, Noguchi D, Chamoto K, Yue Z (2008). IL-6-dependent spontaneous proliferation is required for the induction of colitogenic IL-17-producing CD8+ T cells.. J Exp Med.

[50] Cho JH, Boyman O, Kim HO, Hahm B, Rubinstein MP (2007). An intense form of homeostatic proliferation of naive CD8+ cells driven by IL-2.. J Exp Med.

[51] Schorle H, Holtschke T, Hunig T, Schimpl A, Horak I (1991). Development and function of T cells in mice rendered interleukin-2 deficient by gene targeting.. Nature.

[52] Vezys V, Yates A, Casey KA, Lanier G, Ahmed R (2009). Memory CD8 T-cell compartment grows in size with immunological experience.. Nature.

[53] Selin LK, Lin MY, Kraemer KA, Pardoll DM, Schneck JP (1999). Attrition of T cell memory: selective loss of LCMV epitope-specific memory CD8 T cells following infections with heterologous viruses.. Immunity.

