# Supplementary figures and images for: Cell-to-Cell Interactions and Signals Involved in the Reconstitution of Peripheral CD8+ TCM and TEM Cell Pools
Source: PLoS One. 2011 Mar 14;6(3):e17423. doi: 10.1371/journal.pone.0017423 (PMC3056718; doi:10.1371/journal.pone.0017423)

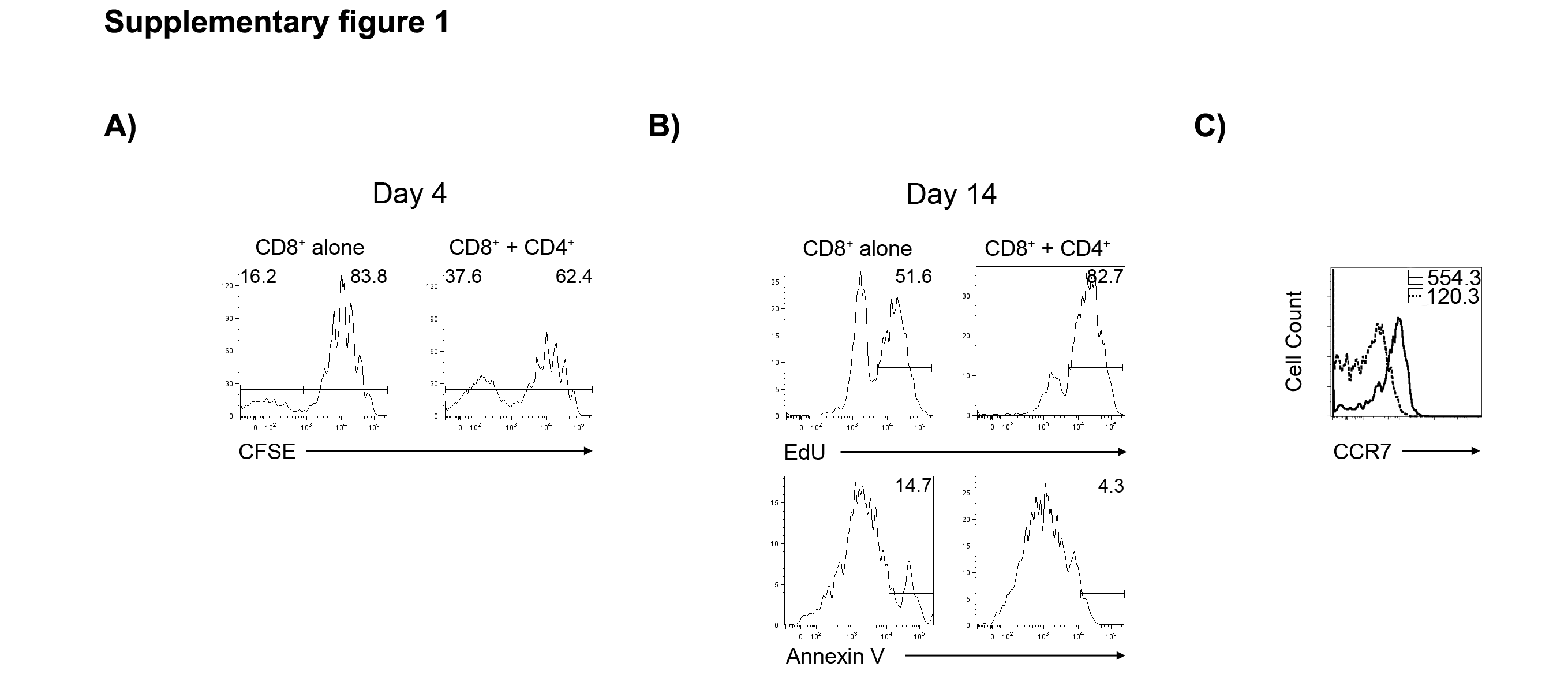

Supplement: Figure S1 — Effects of CD4-help on CD8 T cell division and death rates. (A) CD8+ T cells were stained with CFSE and 106 injected alone or with 2×10^4^ CD4+ T cells into CD3ε−/− mice. The CFSE staining of the CD8+ T cells recovered by day 4 is shown. The percentage of CFSE+ and CFSE− cells is shown in the respective gate. Similar results were obtained in a second independent experiment. (B) 2×10^4^ CD8+ T cells were injected alone or with CD4+ T cells into CD3ε−/− mice. After 10 days, host mice were treated with EdU. Three days latter cells were stained for EdU and Annexin V expression. The upper histograms show EdU staining among the LN CD8+ T cells for one representative host (out of 5). The fraction of Edu stained cells is shown. The lower histograms show the Annexin V staining among the LN CD8+ T cells for one representative host (out of 3). The fraction of Annexin V stained cells is shown. Similar results were obtained in a second independent experiment. (C) The histograms show the CCR7 expression among the recovered CD8+ T cells. The MFI is provided. CCR7 expression by CD8+ T cells was reduced in presence of the CD4+ T cells (dotted line). (TIF) [file pone.0017423.s001.tif]

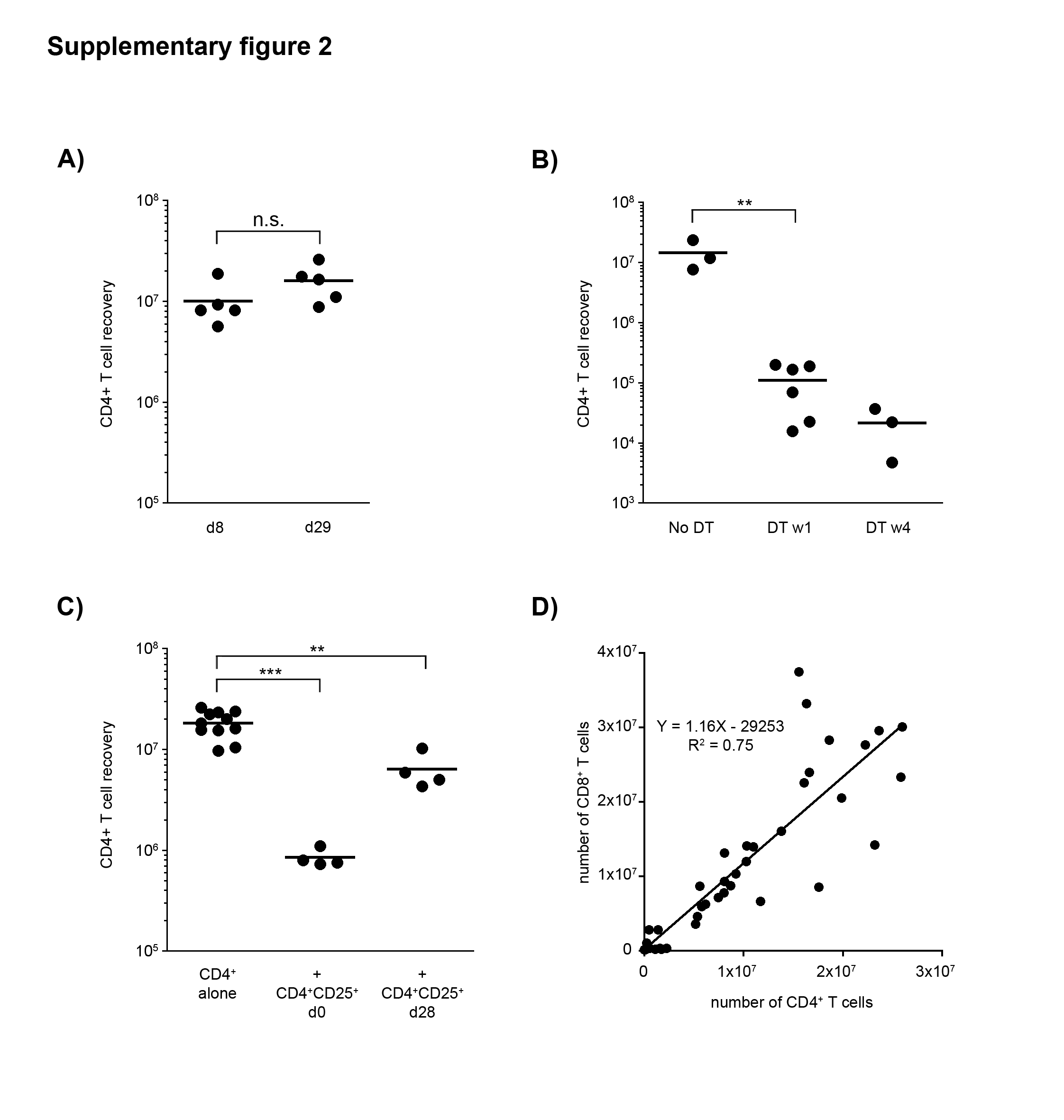

Supplement: Figure S2 — CD4+ T cell recoveries. (A) The absolute number of CD4+ T cells recovered after 2×10^4^ CD4+ T cells were transferred 8 or 29 days after the transfer of 2×10^4^ CD8+ T cells (corresponding to data in Figure 3A). (B) The absolute number of LAT-DTR CD4+ T cells recovered 8 weeks after 2×10^4^ LAT-DTR CD4+ T cells were transferred together with 2×10^4^ CD8+ T cells into host mice that were either left untreated or treated with DT 1 week or 4 weeks after transfer. (C) The absolute number of CD4+ T cells recovered 8 weeks after 104 CD4+ T cells were co-transferred with 2×10^4^ CD8+ T cells, co-transferred with 2×10^4^ CD8+ T cells and 5×10^4^ Treg cells or co-transferred with 2×10^4^ CD8+ T cells into host mice that received 5×10^4^ Treg cells 4 weeks after transfer. (D) Correlation of the number of CD8+ and CD4+ T cells recovered in individual mice from the experiments shown in Figure 3. The correlation coefficients are shown (p<2.6×10^−17^). (TIF) [file pone.0017423.s002.tif]

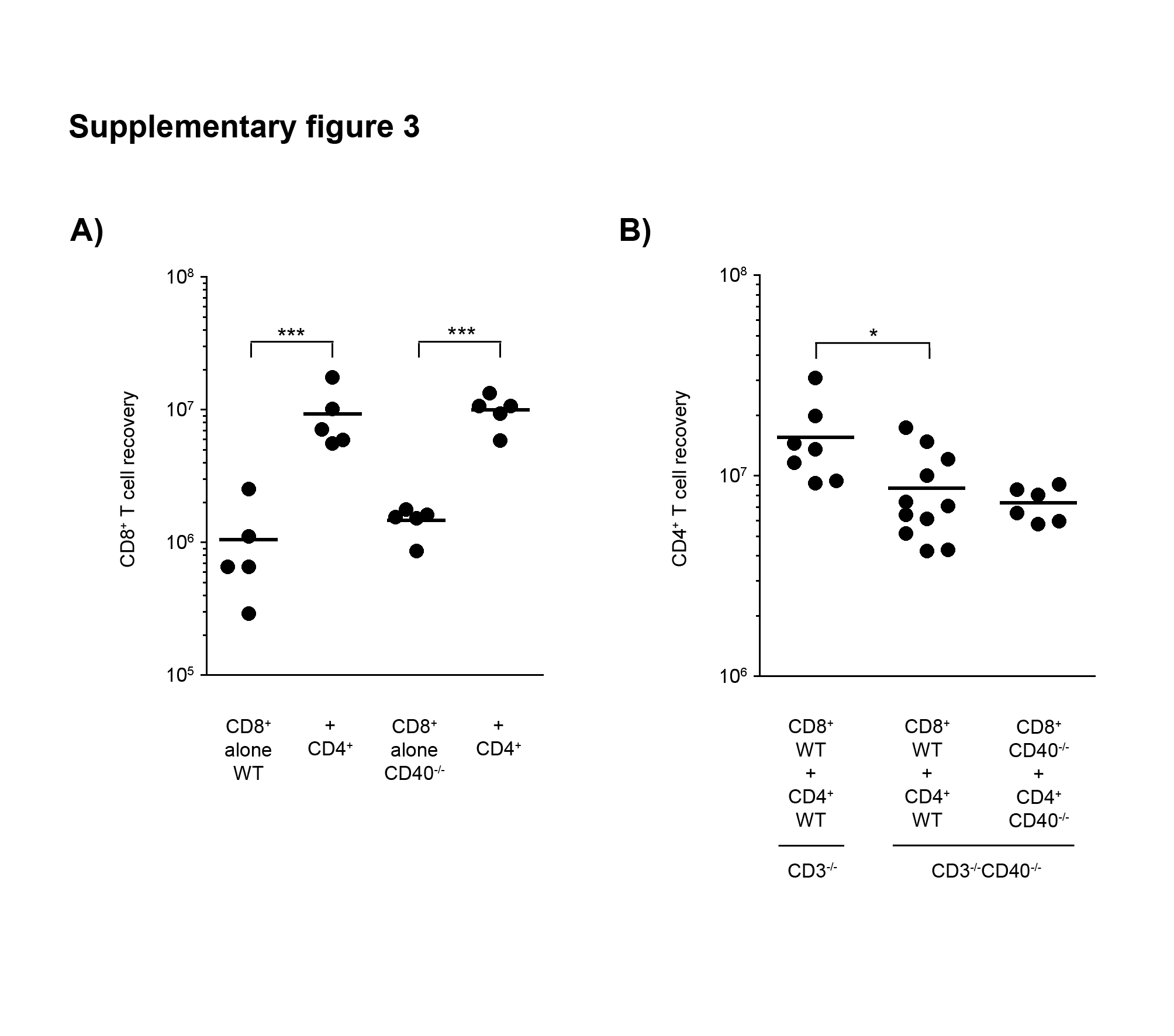

Supplement: Figure S3 — Expansion of CD8+CD40−/− T cells. (A) The absolute number of CD8+ T cells recovered 8 weeks after 2×10^4^ CD8+ T cells from WT or CD40−/− donors were transferred alone or with CD4 T cells into CD3ε−/− mice. The increased accumulation of CD8+ T cells was observed regardless of whether these cells were able to express CD40. (B) Control of the growth of the CD4+ T cells in CD40−/− hosts. CD4+ T cell recovery 10 weeks after the of the transfer of 2×10^4^ CD4+ T cells co-injected with WT or CD40−/− donor T cells into CD3ε−/− or CD40−/−CD3ε−/− hosts. Note that CD4+ T cell expansion still occurred in the absence of CD40. Results are from two pooled experiments. Statistically significant differences are shown (*p≤0.05; ***p<0.001). (TIF) [file pone.0017423.s003.tif]

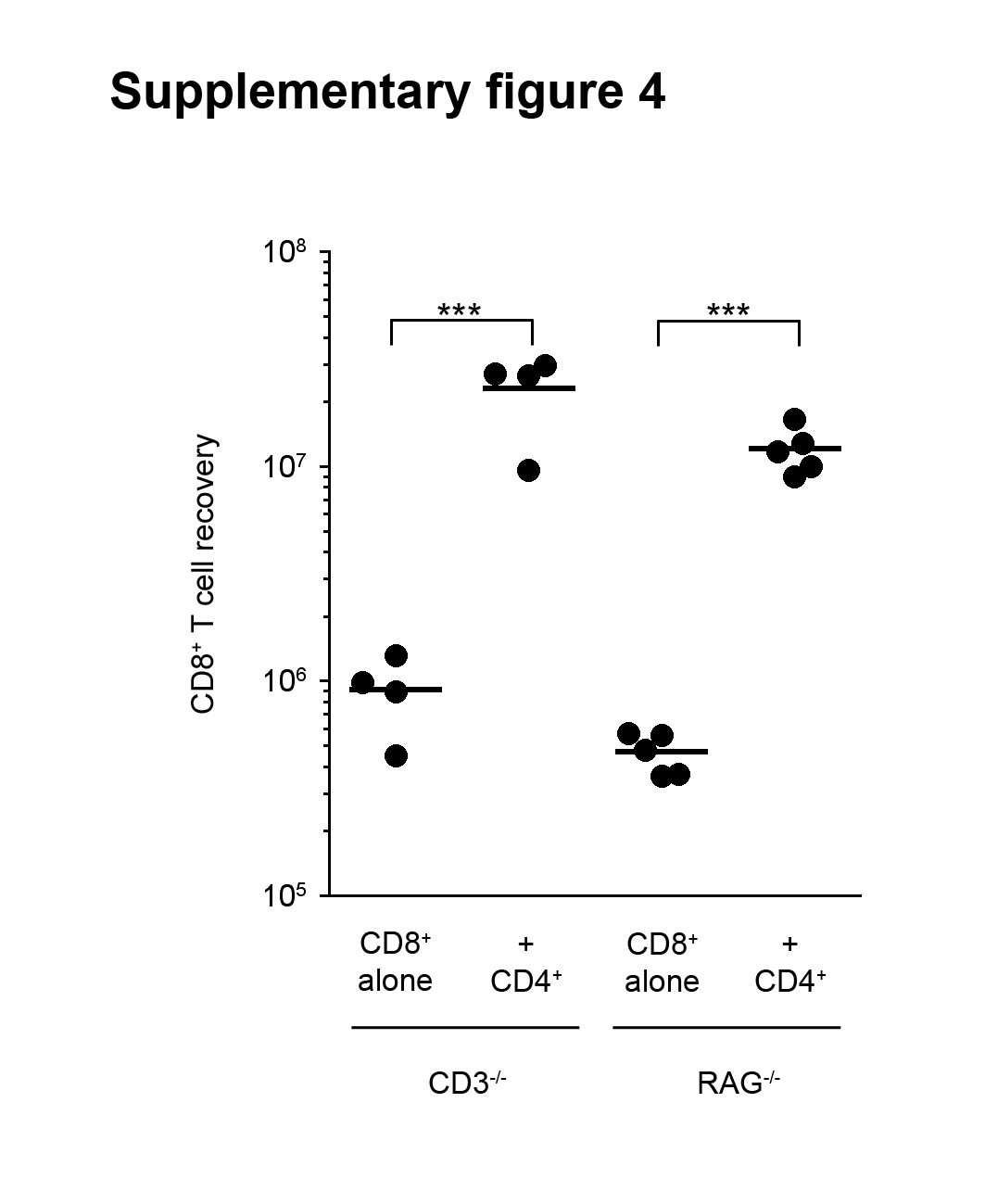

Supplement: Figure S4 — Help in RAG−/− host mice. The absolute number of CD8+ T cells recovered 7 weeks after 2×10^4^ CD8+ T cells were transferred alone or with CD4 T cells into CD3ε−/− or RAG−/− hosts. The helper effect was observed in the presence or absence of B cells. Statistically significant differences are shown (***p<0.001). (TIF) [file pone.0017423.s004.tif]

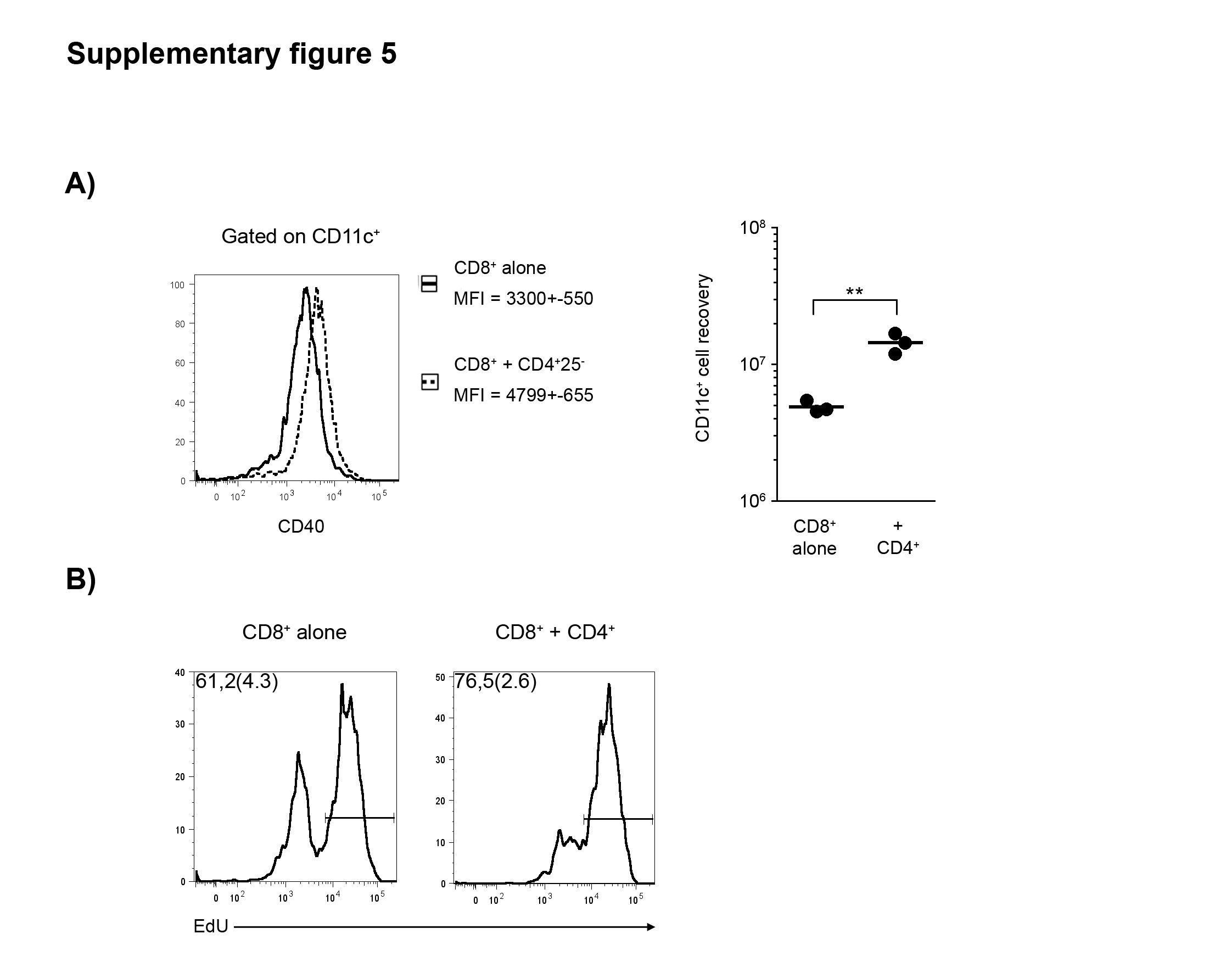

Supplement: Figure S5 — Role of CD11c+ APCs. (A) CD40 expression among the host CD11c+ cells (left) and the total number of host CD11c+ cells (right) recovered 17 days after 2×10^4^ CD8+ T cells were transferred alone or with 2×10^4^ CD4+ T cells into CD3ε−/− mice. (**p≤0.01). Values outside the histogram represent the mean±se (right). To enumerate CD11c+ cells, spleen and LNs were incubated 45 min at 37°C in RPMI containing DNAse I (50 µg/ml) and collagenase type IV (1 mg/ml). In the presence of CD4+ T cells, the number of CD11c+ cells and their CD40 expression increased. CD4+ T cell transfer did not modify CD80, CD86 or MHC class II expression by the host APCs (not shown). (B) The EdU staining among CD11c+ cells in the spleen for one representative host (out of 5). The fraction of Edu stained cells is shown. Similar results were obtained in two independent experiments. (TIF) [file pone.0017423.s005.tif]

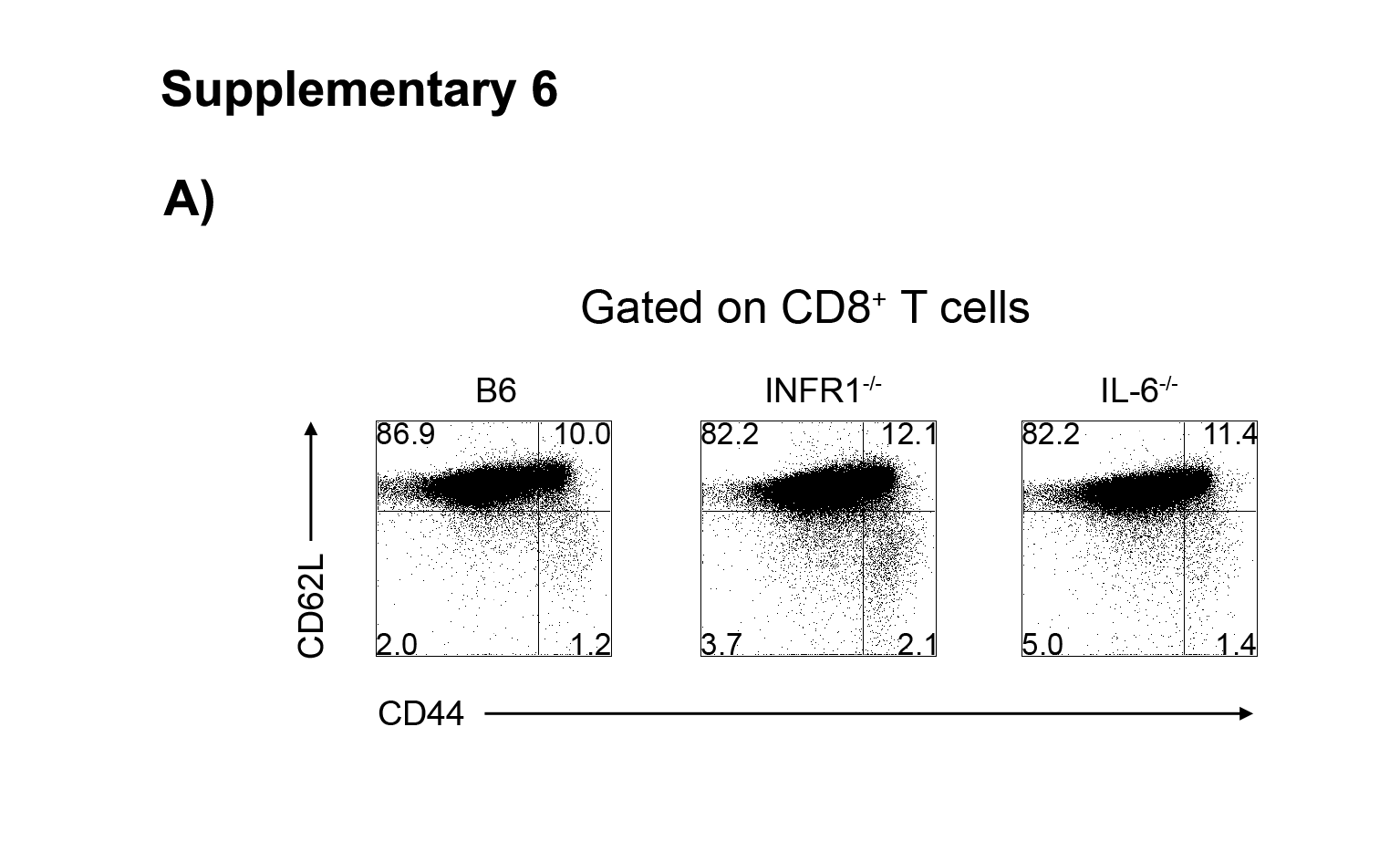

Supplement: Figure S6 — Phenotype of CD8+ T cells. Dot plots show the phenotype of donor CD8+ T cells from WT (left); INFR1−/− (middle) and IL-6−/− mice. (TIF) [file pone.0017423.s006.tif]

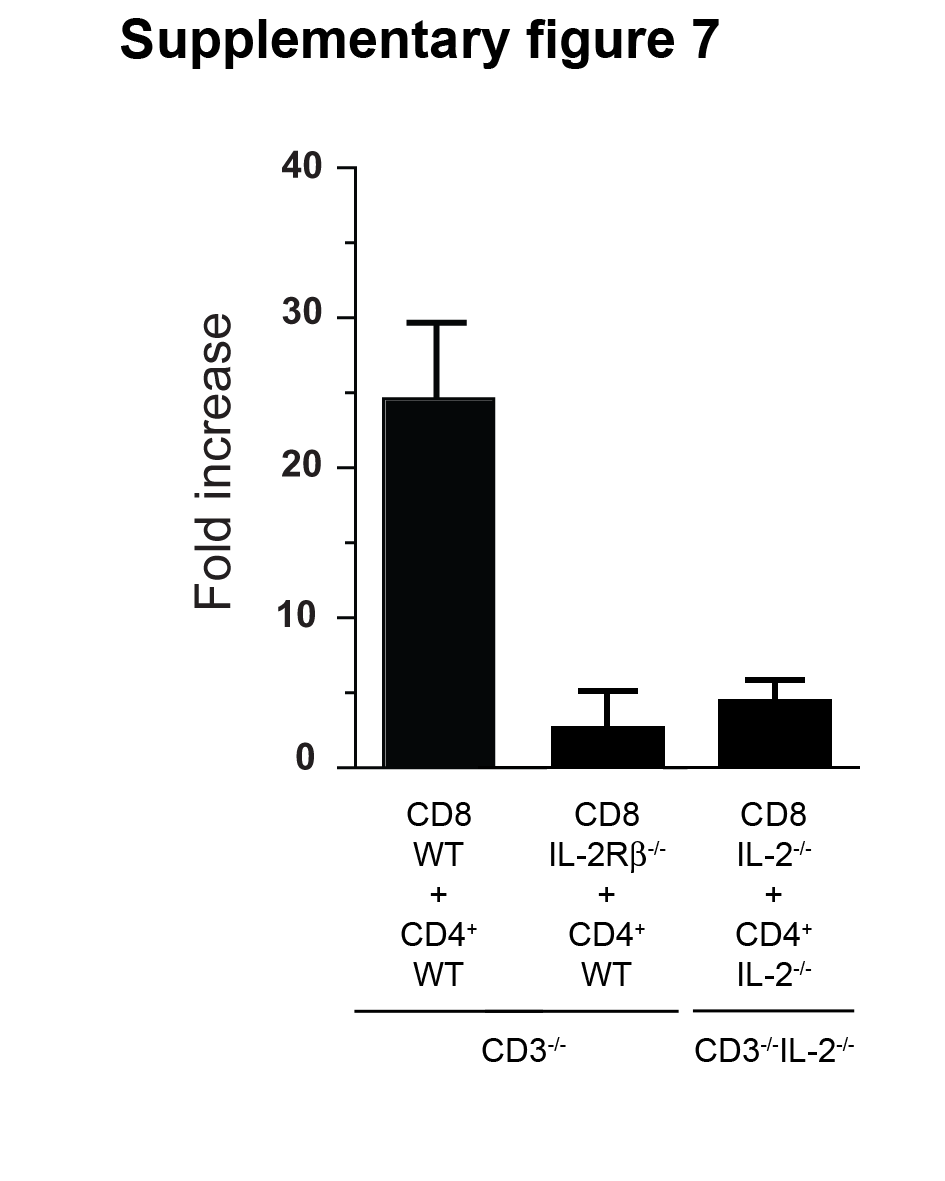

Supplement: Figure S7 — Role of IL-2 in CD4+ T cell help. The fold increases of CD8+ T cell recovery 8 weeks after the transfer of 2×10^4^ CD8+ T cells alone or with 2×10^4^ CD4+ T cells into CD3ε−/− mice, calculated by dividing the number of CD8+ T cells recovered in the presence of CD4+ T cells by the number of CD8+ T cells recovered in the absence of CD4+ T cells. CD8+ T cell recovery increased 10 to 30-fold in the presence of WT CD4+ T cells, but only 2 to 3-fold in the total absence of IL-2 or if they lacked the IL-2Rβ chain. (TIF) [file pone.0017423.s007.tif]
